# Comparison of single-marker and multi-marker tests in rare variant association studies of quantitative traits

**DOI:** 10.1371/journal.pone.0178504

**Published:** 2017-05-31

**Authors:** Stefan Konigorski, Yildiz E. Yilmaz, Tobias Pischon

**Affiliations:** 1 Molecular Epidemiology Research Group, Max Delbrück Center (MDC) for Molecular Medicine in the Helmholtz Association, Berlin, Germany; 2 Department of Mathematics and Statistics, Memorial University of Newfoundland, St. John’s, Newfoundland and Labrador, Canada; 3 Discipline of Genetics, Faculty of Medicine, Memorial University of Newfoundland, St. John’s, Newfoundland and Labrador, Canada; 4 Discipline of Medicine, Faculty of Medicine, Memorial University of Newfoundland, St. John’s, Newfoundland and Labrador, Canada; 5 Charité Universitätsmedizin Berlin, Berlin, Germany; 6 DZHK (German Center for Cardiovascular Research), Berlin, Germany; University Hospital Jena, GERMANY

## Abstract

In genetic association studies of rare variants, low statistical power and potential violations of established estimator properties are among the main challenges of association tests. Multi-marker tests (MMTs) have been proposed to target these challenges, but any comparison with single-marker tests (SMTs) has to consider that their aim is to identify causal genomic regions instead of variants. Valid power comparisons have been performed for the analysis of binary traits indicating that MMTs have higher power, but there is a lack of conclusive studies for quantitative traits. The aim of our study was therefore to fairly compare SMTs and MMTs in their empirical power to identify the same causal loci associated with a quantitative trait. The results of extensive simulation studies indicate that previous results for binary traits cannot be generalized. First, we show that for the analysis of quantitative traits, conventional estimation methods and test statistics of single-marker approaches have valid properties yielding association tests with valid type I error, even when investigating singletons or doubletons. Furthermore, SMTs lead to more powerful association tests for identifying causal genes than MMTs when the effect sizes of causal variants are large, and less powerful tests when causal variants have small effect sizes. For moderate effect sizes, whether SMTs or MMTs have higher power depends on the sample size and percentage of causal SNVs. For a more complete picture, we also compare the power in studies of quantitative and binary traits, and the power to identify causal genes with the power to identify causal rare variants. In a genetic association analysis of systolic blood pressure in the Genetic Analysis Workshop 19 data, SMTs yielded smaller p-values compared to MMTs for most of the investigated blood pressure genes, and were least influenced by the definition of gene regions.

## Introduction

Candidate gene and genome-wide association studies have identified many common variants associated with a large number of complex traits, and explained some of the estimated heritability [1]. The refinement of high-throughput technologies and the decrease in sequencing costs now allow investigating the role of rare variants in greater depth as well. However, despite evidence that many rare variants play a functional role in complex traits and in the regulation of biological processes [2–4], only a relatively small number of rare variants have been found to be associated with complex diseases so far [5–7] with one likely reason that frequently used statistical tests have been underpowered.

While single-marker tests (SMTs) are often the method of choice for the analysis of common and low-frequency single nucleotide variants (SNVs) with a minor allele frequency (MAF) greater than 0.01 or 0.005, multi-marker tests (MMTs) have attracted much attention over the last years for the analysis of rare SNVs. Their development has been motivated by arguments that SMTs have inflated type I error and very low power when testing rare SNVs in the analysis of binary traits [8], and might not provide valid statistical inference when testing very rare SNVs or single base-pair mutations. MMTs use different approaches to combine the rare genetic variants’ information in a given region and test the association of the region with the phenotype. They can be broadly classified as (i) burden tests, which obtain genetic scores by collapsing rare variants in a region/gene [8–11], (ii) extensions of burden tests [12–14], (iii) variance-component tests, which collapse single-variant score statistics in a region [15–17], (iv) combinations of burden and variance-component tests [18–21], and (v) other approaches [22]. Detailed comparisons of rare variant association tests for binary traits [9, 15, 20, 23–24] showed that SMTs based on likelihood ratio tests have inflated type I errors, SMTs based on Score tests, Wald tests, and Fisher’s exact tests have deflated type I errors, and all of these SMTs have much lower power compared to MMTs across most of the considered scenarios. These results have also been picked up by most recent reviews [25–27].

For the analysis of quantitative traits, however, extensive comparisons are lacking and few studies have compared SMTs with MMTs. Those studies that have investigated SMTs and MMTs for quantitative traits [11, 28–31] might seem to partially confirm the results of binary traits, but they actually do not allow valid conclusions regarding which approach has the highest power due to the following reasons. Some studies tested different sets of variants with SMTs and MMTs (SNPs versus SNVs [11], including versus excluding singletons and doubletons [31]) or tested different hypotheses by evaluating the power of identifying causal variants for SMTs but assessed the power of identifying causal regions for MMTs [11, 28, 30]. In another study [29], Saad et al. compared SMTs with MMTs in their power to identify the same genetic locus and found that different methods have higher power in different scenarios. But the results are inconclusive since very few replicates were used in the simulation study and the type I errors were not well-calibrated in their study for SMTs as well as MMTs. Finally, most of the recent studies proposing new MMTs did not compare MMTs with SMTs but only compared the performance of different MMTs [12–14, 16–19, 21–22, 32], or only compared SMTs and MMTs for common SNVs [33–35], which cannot be generalized to the analysis of rare SNVs.

As a result of the inconclusive previous studies, the aim of this study is to provide a comparison of SMTs and MMTs for the analysis of quantitative traits based on a clear formalization of the tested hypotheses for each approach. Consequently, we compare an SMT (Wald-type t-test) with popular MMTs (SKAT [17], SKAT-O [19], and a burden test [10]) for testing the same hypothesis (i.e. the same causal loci) in rare variant association studies of quantitative traits under different scenarios. Without loss of generality, we consider a gene as a causal locus in this study. We show at first that all approaches have valid type I error rates and that in particular, single-marker analyses provide unbiased variant effect estimates and valid statistical inference even when singletons or doubletons are under consideration. Based on extensive simulation studies, we then show that for quantitative traits, SMTs can lead to more powerful or equally-powered association tests for identifying a causal gene (i.e., a gene which includes rare/ very rare causal variants) compared to MMTs in many realistic scenarios. More specifically, SMTs lead to more powerful association tests for identifying causal genes than MMTs when the effect sizes of causal variants in the gene are large, and less powerful tests when causal variants have small effect sizes. For moderate effect sizes, whether SMTs or MMTs have higher power depends on the sample size and percentage of causal SNVs. Sensitivity checks show that this is the case if all rare and non-causal common SNVs in a gene are analyzed, and also if the analysis is restricted to rare SNVs only. The latter comparison mostly influences the results of the burden test. We describe the situations when SMTs and when MMTs have higher power in detail, compare the power for identifying causal rare variants and causal genes using SMTs, and investigate the same scenarios for binary traits to provide a more complete description on which study designs and tests yield the highest power. Finally, we conduct a comparison of SMTs with MMTs in a real data genetic association analysis of systolic blood pressure using the Genetic Analysis Workshop 19 data.

## Material and methods

### Hypotheses tested by single-marker and multi-marker approaches

SMTs and MMTs test different null hypotheses with a different focus. The unit under investigation in MMTs is a region of the genome such as a gene. Accordingly, MMTs are testing whether *a given combination of SNVs in a given gene* (burden-type tests) or *any of the SNVs in a given gene* (variance-component-type tests) is associated with the trait of interest, whereas SMTs are testing whether *a given SNV* is associated with the trait of interest. More formally, for a given gene including *k* SNVs, the tested hypotheses for SMTs are

$$H_{0_{i}}:\;\beta_{i}=0\;\text{vs}.\;H_{A_{i}}:\;\beta_{i}\neq 0\;\text{for}\;i=1,\ldots ,k$$ (1)

where *β*_*i*_ is the effect of the *i*-th SNV on the trait under consideration; and the tested hypotheses for MMTs are

$$H_{0}:\;\alpha =0\;\text{vs}.\;H_{A}:\;\alpha \neq 0$$ (2)

where *α* is the effect of the gene on the trait of interest, which could be a single parameter in burden-type tests, or a vector of parameters for the *k* SNV effects in variance-component-type tests with

$$H_{0}:\;\alpha ={\left(\beta_{1},\ldots ,\beta_{k}\right)}^{T}=0\;\text{vs}.\;H_{A}:\;\beta_{i}\neq 0\;\text{for at least one}\;i.$$ (3)

Hence, if SMTs and MMTs are compared by evaluating the power for identifying a given causal variant (SMT) and a given causal gene (MMT), respectively, any conclusion that one approach is more powerful than the other is questionable since different hypotheses are tested. In fact, within such a comparison of different hypotheses, SMTs are by design of the comparison disadvantaged. In order to compare both approaches fairly, we evaluate SMTs and MMTs in their power for identifying the same genetic locus, and choose a gene as locus. For the SMTs, we therefore test whether any of the rare SNVs within the gene shows a significant association with the trait, accounting for multiple testing, in order to declare that the gene is significantly associated.

In the simulation study including *m* replicates of a gene with multiple SNVs, gene-level power estimates are obtained with

$$\hat{Power}=\hat{P}(Reject\;H_{0}|\;H_{A}\;is\;true)=\frac{number\;of\;significant\;tests\;among\;the\;m\;replicates\;generated\;from\;H_{A}}{m}.$$

For SMTs, this becomes

$$\hat{Power}=\frac{number\;of\;replicates\;including\;at\;least\;one\;significant\;SNV}{m}$$

using an appropriate correction for the testing of multiple SNVs within each gene. This power calculation amounts to the *minP* approach, which was also used in [9] and other studies [15, 20, 24]. If all tests were independent, the Bonferroni correction could be used [8], but since there is often some correlation structure between variants, we additionally used the slightly less conservative Benjamini-Hochberg (BH) correction [36]. We report the power for the nominal significance level of α = 0.05 used in a candidate-gene analysis and of 2.5∙10^−6^ used in a genome-wide analysis of 20,000 genes. Empirical type I error estimates were obtained similarly for data generated from the null hypothesis. If not mentioned otherwise, all results reported in this study regarding type I error rates and power refer to this gene-level evaluation.

For the additional question investigated in this study, how the power of SMTs for identifying a causal gene compares to the power for identifying single causal rare variants, we also obtain SNV-level power estimates for detecting a causal rare SNV with a given MAF, based on the p-values of all *l* SNVs with the given MAF from all replicates. For a nominal *α* level of 0.05, this becomes:

$$\hat{Power}\;=\;\frac{number\;of\;p-values\;\leq 0.05\;among\;the\;l\;SNVs}{l}.$$

### Simulation study

In the simulation study, our aims were to investigate the properties of variant effect estimates obtained through single marker analysis and of the SMT (Wald-type t-test), and to compare the empirical type I error and power estimates of the SMT with popular MMTs (a burden test, SKAT, and SKAT-O) in association studies of quantitative traits. A normally-distributed quantitative trait *Y* was generated given causal SNVs (under the alternative hypothesis scenarios) and two additional covariates. Then, different scenarios varying the percentage of causal variants, their effect sizes, and direction of effects were considered to evaluate the empirical type I error and power of the tests. The set-up of the simulation study is described in more detail in the following.

#### Genetic data generation and simulation study set-up

For the main study, we used the genetic dataset provided in the SKAT package in R [37], which contains 10,000 haplotypes over a 200kb region (including 3845 SNVs) generated from a calibration coalescent model mimicking the LD structure of European ancestry. This was chosen to make our study comparable to the evaluation of SKAT-O in [19]. Accordingly, the kernels and other SKAT options were chosen to reach an optimal performance for the power of SKAT and SKAT-O.

Similar to [19], we randomly sampled 10,000 3kb regions from the 200kb region, to obtain genes with average length. We then randomly paired 2,000 haplotypes of these 10,000 genes to generate *m* = 10,000 replicates with genotypes of *n* = 1,000 individuals for the simulation study. With this, there were on average 33 non-monomorphic SNVs *g*_*i*_ in each replicate (min = 19; max = 52 SNVs), and in total 325,393 SNVs in all *m* = 10,000 replicates combined. Most of the SNVs were rare with MAF≤0.03. In detail, of the 325,393 SNVs, 132,797 SNVs had MAF = 0.0005 (minor allele count MAC = 1); 41,341 SNVs had MAF = 0.001 (MAC = 2); 50,836 SNVs had 0.001<MAF≤0.005 (2<MAC≤10); 39,835 SNVs had 0.005<MAF≤0.03; and 60,584 SNVs had MAF>0.03.

#### Phenotype generation

To evaluate the type I error and the power of the test statistics, we generated *Y* conditional on a binary covariate *x*_*1*_ and a normally distributed covariate *x*_*2*_ (and conditional on the causal SNVs *g*_*i*_ under the alternative hypotheses). We used the same model and parameter values as described in [19] with additive genetic effects:

$$Y=0.5x_{1}+0.5x_{2}+\sum \beta_{i}g_{i}+\varepsilon$$ (4)

with *x*_1_∼*Bin*(0.5), *x*_2_∼*N*(0,1), ε∼*N*(0,1), *β*_*i*_ = *c* ∙ |*log*_10_ (MAF_*i*_)|, where different values of *c* were considered in the simulation study.

For the evaluation of type I error rates, *m* = 10,000,000 replicates were analyzed, and for the power comparisons, *m* = 10,000 replicates were used. We used the sample size of *n* = 1,000 individuals for all simulations. Table 1 gives an overview of all considered scenarios. For the type I error evaluation, empirical gene-level estimates were obtained for all approaches under the null hypothesis given in scenario 0 in Table 1. For the power evaluation, we considered 36 different scenarios, extending the investigation in [19]. They differ in percentages of causal rare variants, effect sizes, and direction of the effects of causal rare variants. Causal variants were randomly chosen among all rare variants with MAF≤0.03. Between 5% and 50% of the rare variants in a gene were set to be causal, and at least 1 rare variant per gene was chosen as causal under the alternative hypothesis. The median of the explained phenotypic variation by all causal SNVs in a gene was less than or equal to 1% in most scenarios, and ranged between 0.1% in scenario 3 (5% causal SNVs in a gene with small effect sizes) and 9.1% in scenario 10 (50% causal SNVs in a gene with larger effect sizes), calculated as the sum of $2∙{MAF}_{i}∙(1-{MAF}_{i})∙\beta_{i}^{2}/Var(Y)$ over all variants *i* in the gene [38].

**Table 1:** Detailed overview of the different scenarios considered for the type I error and power study.

Investigation	Scenario	% of causal variants	Effect size weights	Direction of effect: Positive / Negative	Median (MAD) of explained variance in %
Type I error	0	0%	c = 0	-	-
Power	1	5%	c = 0.6	100% / 0%	0.9% (0.6)
	2	5%	c = 0.3	100% / 0%	0.2% (0.2)
	3	5%	c = 0.2	100% / 0%	0.1% (0.1)
	4	10%	c = 0.6	100% / 0%	1.9% (1.4)
	5	10%	c = 0.3	100% / 0%	0.5% (0.3)
	6	10%	c = 0.2	100% / 0%	0.2% (0.2)
	7	20%	c = 0.6	100% / 0%	3.8% (2.1)
	8	20%	c = 0.3	100% / 0%	1.0% (0.6)
	9	20%	c = 0.2	100% / 0%	0.4% (0.2)
	10	50%	c = 0.6	100% / 0%	9.1% (3.0)
	11	50%	c = 0.3	100% / 0%	2.4% (0.9)
	12	50%	c = 0.2	100% / 0%	1.1% (0.4)
	13–24	As in scenarios 1–12	As in scenarios 1–12	80% / 20%	As in scenarios 1–12
	25–36	As in scenarios 1–12	As in scenarios 1–12	50% / 50%	As in scenarios 1–12

The scenarios vary the percentage of causal variants, their effect size, and the percentage of causal variants with effects in positive/ negative direction. Scenarios 13–24 and 25–36 have the same percentage of causal SNVs and the same effect sizes as scenarios 1–12, but 80% / 20% and 50% / 50% of effects in positive / negative direction. The percentage of causal rare variants is with respect to the total number of rare variants with MAF≤0.03 in the gene. The effect size of a variant with a given MAF on the trait *Y* is *β*_*i*_ = *c* ∙ |log_10_(MAF_*i*_)|. The percentage of explained variance for a given gene is calculated as the sum of $2∙{MAF}_{i}∙(1-{MAF}_{i})∙\beta_{i}^{2}/Var(Y)$ over all variants *i* in the gene. Reported are the median and the median absolute deviation (MAD) of this heritability estimate over the 10,000 replicates.

#### Investigated rare variant tests

To evaluate the performance of SMTs, a linear regression model of *Y*,

$$Y=\gamma_{0}+\gamma_{1}x_{1}+\gamma_{2}x_{2}+\beta_{i}g_{i}+\varepsilon ,\;\varepsilon ∼N\left(0,\sigma^{2}\right)$$ (5)

was separately fitted for each SNV *g*_*i*_ in a gene using the *lm()* function in R, which provides the maximum likelihood estimate $\hat{\beta_{i}}$ of the coefficient *β*_*i*_ and its standard error estimate $\hat{SE}(\hat{\beta_{i}})$ using the unbiased estimate of σ^2^ [39]. Then, the Wald-type t-test statistic $\hat{\beta_{i}}/\hat{SE}(\hat{\beta_{i}})∼t_{n-4}$ was obtained for testing the null hypothesis in (1). In order to account for testing multiple SNVs in a gene in the SMT, we applied the Bonferroni and BH-correction in the type I error evaluation, and the BH-correction in the power estimation.

With respect to multi-marker approaches, three different tests were conducted incorporating all SNVs in a replicate (i.e. gene). The SKAT and SKAT-O were computed using the default linear-weighted kernel (relating the genotypes to the phenotype) in the test statistics as it returned the highest power estimates among all possible options provided in the *SKAT()* function in the *SKAT* package in R. The weights in the weighted kernels of SKAT and SKAT-O are a function of the MAF of SNVs. For the p-value estimation, the default *“davies”* setting was used for SKAT and *“optimal*.*adj”* for SKAT-O. Furthermore, a burden test was conducted by obtaining a gene score as the summation of the minor alleles of all SNVs in the gene (i.e., Σ*g*_*i*_ for each individual, and testing the null hypothesis given in (2) under the linear regression model

$$Y=\gamma_{0}+\gamma_{1}x_{1}+\gamma_{2}x_{2}+\alpha \sum g_{i}+\varepsilon$$ (6)

using the Wald-type t-test statistic obtained from the *lm()* function in R. All common and rare SNVs in the gene were included in the analysis of the SMT and each MMT for a fair comparison. Additional results from analyzing rare variants only are provided in the supplementary materials as a sensitivity analysis. All computations and visualizations were performed in R 2.15.0 and higher.

### Application: Analysis of the genetic analysis workshop 19 data

For a comparison of the SMT and MMTs in a real data application, we performed a gene-level association analysis of systolic blood pressure (SBP) in the Genetic Analysis Workshop 19 data [40–41]. The data stems from the T2D-GENES consortium and contains whole genome-sequence data, blood pressure phenotypes and non-genetic covariates (sex, age, smoking, antihypertensive medication). More specifically, we tested the association of 9 previously identified blood pressure (SBP, diastolic BP, or pulse pressure) genes on chromosome 19 (INSR, RRAS, ZNF101, ELAVL3, RGL3, AMH, DOT1L, PLEKHJ1, SF3A2) [42–45] with SBP, using the complete data available for 103 unrelated individuals. Some individuals received antihypertensive medication so that their observed SBP values were lower than their underlying “true” SBP values. Adjusting SBP for the medication effects is necessary because the objective was to explain the variation in SBP. Hence, following the method in Konigorski et al. [46], we estimated the true SBP values of treated individuals from the conditional expectation of SBP given that the observed SBP is lower than the true SBP and also conditional on the non-genetic covariates. Then, we obtained phenotypes *SBP*_*adj*_ for the analysis, which are adjusted for medication use and non-genetic covariates, as follows. First, we fitted a censored regression model conditional on the non-genetic covariates age, sex, and smoking status, with medication use as a censoring indicator. Then, for an untreated individual, *SBP*_*adj*_ is defined as the difference between observed and fitted SBP from the censored regression model; for a treated individual, *SBP*_*adj*_ is the difference between the estimated true and fitted SBP.

A query to the Ensembl database with reference genome GRCh37.p13 through BioMart was used to define gene regions as 5kb around the gene start and end positions for the gene-level testing. After basic standard quality checks and exclusion of SNVs with more than 5% missing base calls, this yielded 2,779 common (MAF>0.03) and rare (MAF≤0.03) SNVs in the 9 defined gene regions for the gene-level association tests with *SBP*_*adj*_. The SMT, SKAT, SKAT-O, and burden test were computed as described in the Simulation Study section. For SKAT and SKAT-O, the *SKAT()* function in the SKAT R package was used with default settings. For the SMT, a Wald-type t-test was computed under the linear regression model for each SNV, the p-values in a gene were adjusted with the BH-correction, and the minimum adjusted p-value was extracted for each gene for the gene-level association test. First, all 2,779 common and rare SNVs were included leading to gene sizes between 59 and 1,395 SNVs with an average gene size of 309 SNVs. Second, the analysis was restricted to the 1,746 rare SNVs, which led to an average gene size of 194 SNVs.

## Results

### Validity of estimates and test statistics of single-marker analyses

**Fig 1:**
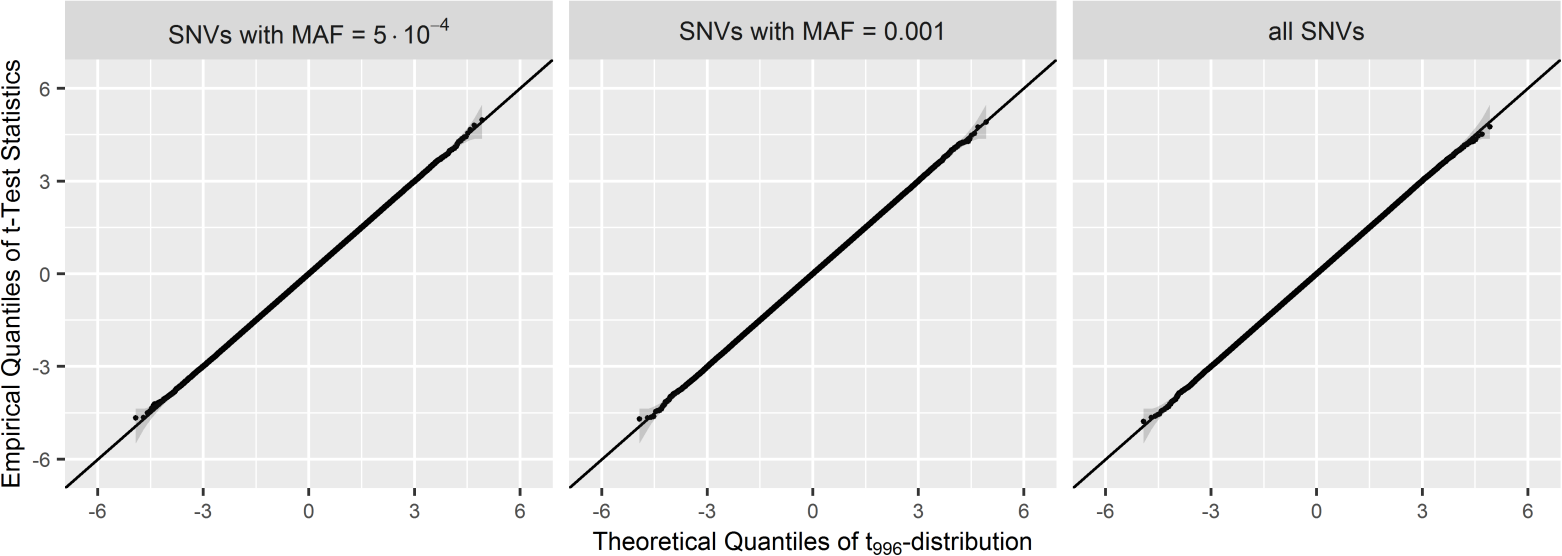
Quantile-quantile plots of SMT statistic values for singletons, doubletons, and all SNVs. Datasets were generated from the null model described in scenario 0 in Table 1 of size *n* = 1,000 for *m* = 10,000,000 replicates. Quantile-Quantile plots are shown comparing the empirical quantiles of the t-test statistics of singletons (left panel), doubletons (middle panel), and all SNVs (right panel) to the theoretical quantiles of the t_df = 1000–4_ distribution. For computational purposes, each plot is based on a random sample of 1,000,000 t-test statistics, out of the 132,797,000 t-test statistics of all singletons in all replicates, out of the 41,341,000 t-test statistics of all doubletons in all replicates, and out of the 325,393,000 t-test statistics of all SNVs in all replicates. In grey ribbons, approximate 95% point-wise confidence intervals are shown.

First, the coefficient estimates of SNVs in linear regression models (5) of quantitative traits and their corresponding standard error estimates were assessed under the null hypothesis (scenario 0 in Table 1) by focusing on singletons only, doubletons only, and all rare SNVs. The results showed that the variant effect estimates are unbiased and that the standard error estimates are equal to the standard deviations of the variant effect estimates, even when singletons and doubletons are under consideration (S1 Table). Investigation of the single-marker t-test statistic for testing the hypotheses in (1) showed that the distribution assumption holds as well for SNVs of all MAF (Fig 1, S1 Fig).

### Evaluation of type I error rates of the SMT and MMTs

Next, the empirical type I errors of the SMT and MMTs for testing the hypothesis in (2) were investigated for different nominal levels, to assess whether they are well-controlled (Table 2). The results indicate that empirical type I errors of all approaches are close to the nominal levels, allowing a valid comparison of the power estimates. For the power evaluations in the following, the BH correction was used for the SMT in order to account for the multiple testing of all SNVs within a gene and obtain gene-level power estimates, since some dependencies were observed between SNVs and since the empirical type I errors were closer to the nominal level compared to using the Bonferroni correction for most of the nominal levels.

**Table 2:** Empirical type I error of MMTs and SMT for different nominal *α* levels.

	MMTs			SMT	
Nominal *α*	SKAT	SKAT-O	Burden	Linear Regression Bonferroni correction	Linear Regression BH correction
5 ∙ 10^−2^	4.94 ∙ 10^−2^	5.22 ∙ 10^−2^	5.01 ∙ 10^−2^	4.39 ∙ 10^−2^	4.93 ∙ 10^−2^
1 ∙ 10^−2^	0.97 ∙ 10^−2^	1.10 ∙ 10^−2^	1.00 ∙ 10^−2^	0.90 ∙ 10^−2^	0.99 ∙ 10^−2^
1 ∙ 10^−3^	0.94 ∙ 10^−3^	1.12 ∙ 10^−3^	1.00 ∙ 10^−3^	0.88 ∙ 10^−3^	0.97 ∙ 10^−3^
1 ∙ 10^−4^	0.92 ∙ 10^−4^	1.17 ∙ 10^−4^	1.03 ∙ 10^−4^	0.91 ∙ 10^−4^	0.98 ∙ 10^−4^
1 ∙ 10^−5^	0.92 ∙ 10^−5^	1.10 ∙ 10^−5^	1.12 ∙ 10^−5^	1.16 ∙ 10^−5^	1.25 ∙ 10^−5^
2.5 ∙ 10^−6^	2.40 ∙ 10^−6^	2.40 ∙ 10^−6^	3.50 ∙ 10^−6^	2.40 ∙ 10^−6^	2.90 ∙ 10^−6^

Data was generated from the null model with size *n* = 1,000 for *m* = 10,000,000 replicates.

As a sensitivity check, misspecified distributions for the error term in model (5) were considered. Data was generated from the null model in (4) with error terms from the t-distribution with 4 and 8 degrees of freedom and from the standard log-normal distribution. Then, the SMT, SKAT, SKAT-O and burden tests were conducted to test the absence of genetic effects assuming normally-distributed errors as described in the Materials and Methods section. The results are shown in S2 Table and indicate that only the burden test has valid type I errors. The SKAT, SKAT-O and SMT are all sensitive to a misspecification of the error distribution, and the SMT shows a much higher inflation of empirical type I errors. The inflation can be slightly decreased by excluding SNVs with only 1 or 2 observed minor alleles but is still substantial and much higher than of SKAT and SKAT-O.

### Power for identifying a causal gene with the SMT and MMTs

**Fig 2:**
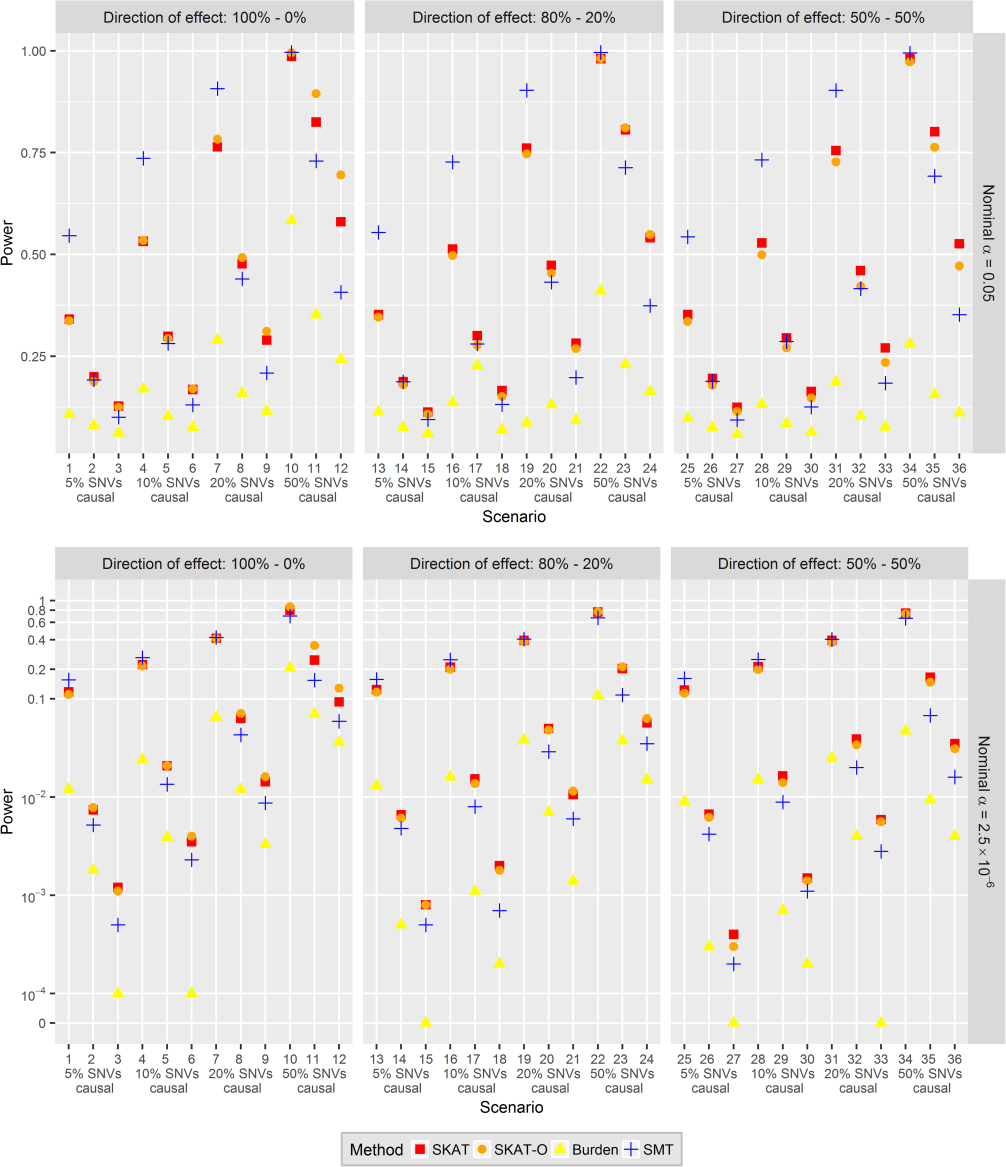
Power estimates of the SMT and MMTs. Data was generated under an alternative-hypothesis model described in scenarios 1–36 in Table 1 of size *n* = 1,000 for *m* = 10,000 replicates. The nominal *α* was set to 0.05 (upper panel) and 2.5∙10^−6^ (lower panel). In the lower panel with *α* = 2.5∙10^−6^, the coordinate system is shown on a log_10_-scale to better visualize the small power differences between the approaches. Multiple testing corrections for the SMT of all SNVs in a gene were done using the BH-correction.

Fig 2 and S3 and S4 Tables show the power estimates for all test statistics under the scenarios 1–36 described in Table 1, when the type I error was 0.05 or 2.5∙10^−6^. The burden test had the lowest power among all tests in all scenarios when all rare and (non-causal) common variants were included in the analysis. Regarding a comparison among the other MMTs, SKAT-O had generally similar power with SKAT and the only noticeable differences were in scenarios 11, 12 (50% causal rare SNVs, all effects in the same direction) when the power of SKAT-O was 7–11.5% higher for α = 0.05 and 3.5–10% higher for α = 2.5∙10^−6^. These results are in line with the literature [19].

Regarding the comparison of the SMT with MMTs, a first observation was that the power differences between the SMT and MMTs were very small for a genome-wide scan (*α* = 2.5∙10^−6^), and more pronounced for candidate-gene testing (*α* = 0.05) when using a sample size of *n* = 1,000. The same tendencies held for both nominal significance levels, and the further most important determinants of which test has higher power were the effect size and the percentage of causal variants. As main results, the SMT had consistently the highest power when the effect sizes were higher (*c* = 0.6), for all percentages of causal SNVs and both nominal significance levels (except when there were 50% causal SNVs in a gene and *α* = 2.5∙10^−6^). When the effect sizes were moderate (*c* = 0.3) and small (*c* = 0.2), the power of SMTs and MMTs were very similar when there were 5% or 10% causal SNVs, SKAT/SKAT-O had slightly higher power when there were 20%, and larger power when there were 50% causal SNVs. For a given effect size, increasing the percentage of causal variants in a gene led to a power increase for each test. The direction of SNV effects within a gene did not seem to greatly influence the question which test has the highest power, illustrating the robustness of SKAT/SKAT-O.

For an illustration and explanation of the performance of the SMT, we provide more details for the main results of scenario 4 in the following. Here, the SMT had a power of 26.3% to identify a causal gene in a genome-wide study under the nominal *α* level of 2.5∙10^−6^. That means that within 2,629 replicates (26.3% of the 10,000 replicates), there was at least one rare SNV in a replicate (gene) with adjusted p-value smaller than 2.5∙10^−6^, which led to the identification of the gene as causal. In fact, 3,084 SNVs had adjusted p-value smaller than 2.5∙10^−6^ in the 2,629 replicates: 20 (of 15,108) causal singletons were correctly identified, 14 (of 4,675) causal doubletons were correctly identified, 341 (of 5,874) causal SNVs with MAF between 0.001 and 0.005, 428 (of 1,550) causal SNVs with MAF between 0.005 and 0.01, and 2,281 (of 2,989) causal SNVs with MAF between 0.01 and 0.03. If singletons and doubletons had been excluded from the analysis of linear regression, the power would have been slightly elevated to 28.0% in this scenario. On the other hand, for the candidate-gene approach using a significance threshold of 0.05, the gene-level power of the SMT was 73.6% and substantially more very rare variants were identified and drove the high gene-level power. In more detail, 2,576 (of 15,108) causal singletons were correctly identified, 1,551 (of 4,675) causal doubletons were correctly identified, 3,757 (of 5,874) causal SNVs with MAF between 0.001 and 0.005, 1,482 (of 1,550) causal SNVs with MAF between 0.005 and 0.01, and 2,980 (of 2,989) causal SNVs with MAF between 0.01 and 0.03. For this analysis, the power would have been substantially decreased to 62.4% by excluding singletons and doubletons.

We conducted several sensitivity checks to add further details to the comparison. First, phenotype data was generated for scenarios 1–12 under the alternative hypothesis using the same parameter values as in Table 1, but for larger sample sizes and based on larger genes. More specifically, we considered the same genetic dataset and the same percentage of causal SNVs but with on average 58 SNVs or 572 SNVs per gene with a sample size of *n* = 5,000. Also, another genetic dataset (‘SKAT.example’ data in the SKAT R package) including 67 SNVs per gene was used to generate data under scenarios 1, 4, 7, 8, 12 with a sample size of *n* = 2,000. The power estimates are shown in S5–S7 Tables, respectively, and increased naturally for all methods. When a gene of average size 33 SNVs with a sample size of *n* = 1,000 was analyzed in comparison to a gene with average size 58 SNVs with a sample size of *n* = 5,000, the increased sample size helped SMTs with a higher power increase compared to MMTs. For example, while the power of the SMT versus SKAT-O was 19.2% versus 19.9% (for *α* = 0.05) and 0.5% versus 0.7% (for *α* = 2.5∙10^−6^) in scenario 2 with *n* = 1,000 (S3 and S4 Tables), it was 19.5% versus 15.3% (*α* = 2.5∙10^−6^) for the larger sample and gene size. Hence, for larger sample sizes, SMTs are more powerful than all MMTs also for moderate effect sizes. These conclusions did not change when the same larger sample size (*n* = 5,000) was analyzed with much larger genes including on average 572 SNVs (S6 Table). The results of analyzing a sample size of *n* = 2,000 and a gene size of 67 yielded similar conclusions compared to the main analysis including *n* = 1,000 individuals.

As a second sensitivity check, we conducted the analysis under the alternative scenarios by only analyzing rare SNVs (with MAF≤0.03) in a gene and disregarding the (non-causal) common SNVs. The power of all approaches increased, but the conclusion which approach has the highest power in each scenario did not change, for both nominal levels (see S3 and S4 Tables). As a notable difference, the power of the burden test was closer to the power of SKAT and SKAT-O, which is in line with the results reported in [19]. Furthermore, the power increase of SKAT and SKAT-O was marginally higher than of the SMT. Third, if the more conservative Bonferroni correction is used to account for testing multiple SNVs in a gene with SMTs, the power decreases slightly, but does not change any of the conclusions regarding which method has the highest power in which scenario (see S8 Table).

In addition, the same simulation set-up was used to generate a case-control trait from a logistic regression model to compare the SMTs and MMTs for an analysis of a binary trait. Further details are given in S1 Text, and results of the power comparison in S9 and S10 Tables. The results indicate that the power of all tests was much lower compared to the analysis of quantitative traits. The decrease was much stronger for the SMT, which had a very low power across all scenarios in line with previous studies [15, 20, 24].

### Power for identifying a causal variant versus a causal gene

After comparing the single-marker and multi-marker approaches in their ability to identify a causal gene, another important question for practical applications is: What is the power of the tests for identifying a causal variant for a quantitative trait, and how does it compare to the power for identifying a gene containing causal SNVs (i.e., causal gene)? Importantly, this question should not be confused with a power comparison of SMTs (which can be used to identify both causal genes and causal variants) and MMTs (which can be used to identify causal genes). Obtaining SNV-level power estimates for SMTs is straightforward, and S11 Table shows power estimates of SMTs for identifying a causal single variant with a given MAF in comparison to the power for identifying a causal gene (as shown in Fig 2). For variants with low MAF (e.g. MAF<0.005), the power for identifying a single variant is very low relative to gene-level tests across all scenarios, which corresponds to the reported low power of SMTs in the literature. For SNVs with MAF≥0.005, the power of variant-level tests is higher than the power of gene-level tests when there are 5% causal SNVs in a gene, and it is again lower compared to gene-level tests when there are higher percentages of causal SNVs. More details and additional results are presented in S2 Text and S12 Table.

### Genetic association analysis of blood pressure genes

In the genetic association analysis of the adjusted SBP phenotype, gene-level hypothesis tests were performed using the SMT, SKAT, SKAT-O, and the burden test. The normal distribution assumption for the error terms in the linear regression of the adjusted SBP phenotype was satisfied. The p-values obtained under each test to assess the significance of the 9 candidate genes are shown in Table 3, for the analyses based on two different inclusion criteria of SNVs: all common and rare SNVs in each gene, and only rare SNVs. The comparison showed that the SMT yielded the smallest p-value for most candidate genes, and was the only approach with a p-value less than 0.05. Furthermore, the power estimates of the SMT, SKAT, and SKAT-O are relatively consistent using either inclusion criterion for SNVs. The burden test, on the other hand, is more strongly influenced by the inclusion criterion.

**Table 3:** Gene-level p-values for the association tests of candidate genes with SBP in the genetic analysis 19 data analysis.

Gene	SKAT		SKAT-O		Burden		SMT	
	All SNVs	Rare SNVs	All SNVs	Rare SNVs	All SNVs	Rare SNVs	All SNVs	Rare SNVs
INSR	0.51	0.73	0.72	0.76	0.27	0.70	0.14	0.07
RRAS	0.33	0.32	0.49	0.46	0.59	0.34	0.26	0.18
ZNF101	0.94	0.93	0.91	0.87	0.97	0.72	0.98	0.97
ELAVL3	0.46	0.44	0.29	0.42	0.12	0.27	0.76	0.74
RGL3	0.21	0.13	0.32	0.22	0.46	0.22	0.03	0.02
AMH	0.30	0.24	0.46	0.38	0.65	0.73	0.18	0.12
DOT1L	0.59	0.60	0.78	0.79	0.43	0.73	0.70	0.51
PLEKHJ1	0.59	0.50	0.79	0.72	0.33	0.67	0.18	0.13
SF3A2	0.50	0.36	0.70	0.54	0.41	0.97	0.23	0.15

Unadjusted p-values from gene-level genetic association analysis of all common and rare SNVs (“all SNVs”), and only rare SNVs (“rare SNVs”) in 9 candidate genes with SBP. Adjustments for multiple testing of all SNVs in SMTs in a gene were done using the BH-correction.

When the analysis was extended to test all 2,858 gene regions on chromosome 19 including 273,636 common and rare SNVs and 218,198 rare SNVs, the same above conclusions were reached but none of the approaches yielded a significant gene-level test for a nominal level of 0.05 corrected for multiple testing.

## Discussion

In this study, we focused on the association analysis of rare variants with quantitative traits and first showed that standard restricted maximum likelihood estimation in a single-marker approach does provide unbiased estimates and single-marker Wald-type t-tests allow valid statistical inference even for very rare SNVs. In the following main analysis, we compared the power of the SMT and MMTs for identifying a gene that includes causal rare variants, and demonstrated that SMTs or MMTs can be more powerful depending on the scenario. More specifically, SMTs lead to more powerful association tests for identifying causal genes than MMTs when the effect sizes of causal variants in the gene are large, and less powerful tests when causal variants have small effect sizes. For moderate effect sizes, whether SMTs or MMTs have higher power depends on the sample size and percentage of causal SNVs. In our opinion, the most realistic scenarios are that at most 5% or 10% rare SNVs in a gene are causal. Then, for moderate effect sizes, the SMT has higher power when the sample size is larger, and slightly less power for a smaller sample size. These results seem to hold consistently for different gene sizes, different nominal *α* levels, different proportions of SNVs with positive/negative effect on the trait, and different corrections for multiple testing of SNVs in a gene (Bonferroni and BH correction) with SMTs. In the data application, the SMT provided the smallest p-values in the testing of previously identified candidate blood pressure genes.

For any gene-level investigation, a very important question is which SNVs should be grouped together in a test. Including more true causal SNVs by extending the region under consideration or by analyzing both common and rare SNVs can increase the power. At the same time, including more non-causal SNVs adds noise and decreases the power. Accordingly, in the simulation study, the power of all approaches increased when the analysis was restricted to rare SNVs, since only rare SNVs were chosen as causal SNVs in the data generation. The same was observed in the real data association study–but only consistently for the SMT–where the reason might have been as well that there were not any common causal SNVs. In the presence of causal common SNVs, including them in gene-level tests should increase the power. The data application also showed that while the SMT, SKAT, and SKAT-O are quite robust against which sets of SNVs are investigated, the burden test is highly sensitive and can lead to vastly different results.

It should be noted that in the simulation study, the data was generated from the same model as in [19] where the effect size of a SNV depends on the MAF of SNVs through a specified function (see Table 1). Since the weighted kernel functions in SKAT and SKAT-O are dependent on the MAF of SNVs as well, this gives SKAT and SKAT-O an advantage and allowed us to evaluate and compare the SMT in situations where the MMTs have high power. Therefore, in real applications, the power of SMTs might compare even more favorably to these MMTs.

Our proposition to use SMTs for the analysis of rare variants is based on the fact that maximum likelihood estimation returns valid estimates under the linear regression model, even for the analysis of singletons and doubletons. Importantly, as described in the Results section, the strategy to include singletons and doubletons in the analysis can substantially improve the power of SMTs. Of course, this does not alleviate the need to check for genotyping errors and other biases, which are especially relevant to singletons and doubletons. Also, while all investigated approaches rely on normality assumptions, the Wald-type t-test (SMT) was much more vulnerable to a distribution misspecification than the MMTs. When the residuals in the genetic association test are not normally distributed for one or multiple SNVs (or when effects follow another genetic model), adapting the test statistic is warranted. Further analyses of our study (data not shown) confirmed that for binary outcomes, standard statistical tests such as Fisher’s exact test, logistic regression likelihood ratio tests, Wald tests, and Score tests, should not be directly used for testing singletons and doubletons since they do not allow valid inference. Therefore, for each test, all assumptions including the (asymptotic) distribution assumptions and empirical type I errors have to be assessed before the analysis, and significant test results have to be cautiously investigated, especially if they are based on singletons or doubletons.

Secondary results of our study provide suggestions regarding the study design of rare variant association studies. We observed that the power to identify causal low-frequency SNVs is almost always much smaller compared to the power to identify gene regions encompassing causal rare SNVs (S11 Table; cf. [28]), except when very few SNVs in a gene are causal, have higher MAF (i.e. MAF between 0.005 and 0.03) and higher effect sizes. This should be kept in mind but not be considered as a power comparison between MMTs and SMTs. Hence, the power of rare variant tests depends highly on which hypothesis is tested, the number of causal SNVs, their effect size, and whether the ultimate goal is to identify a causal gene or a causal SNV. The comparison between SNV-level and gene-level association tests might also depend on the ratio of the number of genes to the number of SNVs, which could be further investigated in future studies. Regarding MMTs, the results come with a loss in information because the exact position of causal variants is not revealed, and to our knowledge, the currently available methodology does not allow to obtain power estimates for identifying a causal SNV. If the focus of the analysis is on identifying causal rare SNVs instead of genes, SMTs can be used in a genome-wide scan for variants, or alternatively SMTs/MMTs can be used in the first stage to identify target genes and followed up by SMTs of variants within these genes (S12 Table). Comparisons between such 1-stage and 2-stage approaches were not the aim of this study, and could be an interesting avenue for future research by incorporating correct adjustments for multiple testing, the 2-stage testing and biases such as the winner’s curse [47]. For choosing the most powerful statistical test for specific situations in genetic association studies of rare variants, the locus of interest (gene or variant) should be specified first, followed then by a comparison of different statistical tests.

Our results add more details and shed a light on the comparison of single-marker and multi-marker tests for quantitative traits, and provide clear suggestions under which assumptions MMTs should be used, and when a simple SMT is favorable. In contrast to popular practice in epidemiological and medical studies, our additional results also highlight that quantitative traits should be analyzed whenever possible instead of binary traits to maximize the power of association tests. It will be interesting to observe if using more powerful tests can help to identify a larger number of functional rare variants in future studies.

## Supporting information

S1 Text Details of the analysis of binary traits. (PDF)

S2 Text Details of the power comparison for identifying a causal variant and a causal gene. (PDF)

S1 Table Descriptive statistics of (restricted) maximum likelihood estimates from linear regression model under the null hypothesis. Datasets were generated from the null model described in scenario 0 in Table 1 with size *n* = 1,000 for *m* = 10,000,000 replicates. The table shows the mean and standard deviation of the SNV effect estimates and the mean of the corresponding standard error estimates, for the 132,797,000 singletons in all replicates, the 41,341,000 doubletons in all replicates, and the 2,985,000 SNVs with 10 observed minor alleles (minor allele count MAC = 10).
(PDF)

S2 Table Empirical type I error estimates of the SMT and MMTs for the nominal level *α* = 0.05 when the distribution of the phenotype is misspecified. Data was generated from the null model with size *n* = 1,000 for *m* = 10,000,000 replicates, with the error distribution of the phenotype given covariates chosen to be from a standard normal (cf. Table 1), t_4_, t_8_, or log standard normal distribution. All approaches were computed as described in the main manuscript assuming normally distributed error terms. Adjustments for multiple testing of all SNVs in a gene with the SMT were done using the BH and Bonferroni corrections. The type I error estimates are based on analyses using all rare and (non-causal) common SNVs in a gene, as well as restricting the analysis to SNVs with at least 2 (MAC>1) or 3 (MAC>2) observed minor alleles for the SMT. For other nominal levels (e.g. 10^−2^, 10^−3^, 10^−4^, 10^−5^, 2.5∙10^−6^, SKAT, SKAT-O and the SMT showed similar results and inflated type I errors, with a much higher inflation for the SMT.
(PDF)

S3 Table Power estimates of the SMT and MMTs under the nominal *α* level of 0.05. Data was generated under the alternative-hypothesis model described in scenarios 1–36 in Table 1 with size *n* = 1,000 for *m* = 10,000 replicates. The nominal *α* level was set to 0.05. Adjustments for multiple testing of all SNVs in a gene with the SMT were done using the BH correction. The power results are provided for analyses using all rare and (non-causal) common SNVs in a gene, and for using all rare SNVs in a gene by excluding the common SNVs from the analysis.
(PDF)

S4 Table Power estimates of the SMT and MMTs under the nominal *α* level of 2.5∙10^−6^. Data was generated under the alternative-hypothesis model described in scenarios 1–36 in Table 1 with size *n* = 1,000 for *m* = 10,000 replicates. The nominal *α* level was set to 2.5∙10^−6^. Adjustments for multiple testing of all SNVs in a gene with the SMT were done using the BH correction. Power results are provided for analyses using all rare and (non-causal) common SNVs in a gene, and for using all rare SNVs in a gene by excluding the common SNVs from the analysis.
(PDF)

S5 Table Power estimates of the SMT and MMTs under the nominal *α* level of 2.5∙10^−6^, for a sample size of 5,000 and genes including 58 SNVs on average. Data was generated under the alternative-hypothesis model described in scenarios 1–12 in Table 1 with size *n* = 5,000 for *m* = 10,000 replicates. The genes analyzed in the replicates included on average 58 SNVs. The nominal *α* level was set to 2.5∙10^−6^. Adjustments for multiple testing of all SNVs in a gene with the SMT were done using the BH correction. Power results are provided for analyses using all rare and (non-causal) common SNVs in a gene, and for using all rare SNVs in a gene by excluding the common SNVs from the analysis.
(PDF)

S6 Table Power estimates of the SMT and MMTs under the nominal *α* level of 2.5∙10^−6^, for a sample size of 5,000 and genes including 572 SNVs on average. Data was generated under the alternative-hypothesis model described in scenarios 1–12 in Table 1 with size *n* = 5,000 for *m* = 10,000 replicates. The genes analyzed in the replicates included on average 572 SNVs. The nominal *α* level was set to 2.5∙10^−6^. Adjustments for multiple testing of all SNVs in a gene with the SMT were done using the BH correction. Power results are provided for analyses using all rare and (non-causal) common SNVs in a gene, and for using all rare SNVs in a gene by excluding the common SNVs from the analysis.
(PDF)

S7 Table Power estimates of the SMT and MMTs under the nominal *α* level of 2.5∙10^−6^, for a sample size of 2,000 and genes including 67 SNVs. As genetic data, the available genotypes of the 67 SNVs of *n* = 2,000 individuals in the ‘SKAT.example’ data in the SKAT R package were used in each replicate. Data was generated under the alternative-hypothesis model described in scenarios 1, 4, 7, 8, 12 in Table 1 with size *n* = 2,000 for *m* = 10,000 replicates. The nominal *α* level was set to 2.5∙10^−6^. Adjustments for multiple testing of all SNVs in a gene with the SMT were done using the BH correction. Power results are provided for analyses using all rare and (non-causal) common SNVs in a gene.
(PDF)

S8 Table Power estimates of the SMT and MMTs under the nominal *α* level of 2.5∙10^−6^ to compare the BH and Bonferroni correction for SMTs. Data was generated under the alternative-hypothesis model described in scenarios 1–12 in Table 1 with size *n* = 1,000 for *m* = 10,000 replicates. The nominal *α* level was set to 2.5∙10^−6^. Adjustments for multiple testing of all SNVs in a gene with the SMT were done using the BH or the Bonferroni correction. Power results are provided for analyses using all rare and (non-causal) common SNVs in a gene.
(PDF)

S9 Table Power estimates of the SMT and MMTs for analyzing a binary trait under the nominal *α* level of 0.05. Case-control data was generated under the alternative-hypothesis model described in scenarios 1, 4, 7, 8, 12 in Table 1 with size *n* = 1,000 for *m* = 10,000 replicates. The nominal *α* level was set to 0.05. Adjustments for multiple testing of all SNVs in a gene with the SMT were done using the BH correction. All rare and (non-causal) common SNVs were incorporated in the analysis.
(PDF)

S10 Table Power estimates of the SMT and MMTs for analyzing a binary trait under the nominal *α* level of 2.5∙10^−6^. Case-control data was generated under the alternative-hypothesis model described in scenarios 1, 4, 7, 8, 12 in Table 1 with size *n* = 1,000 for *m* = 10,000 replicates for a binary trait. The nominal *α* level was set to 2.5∙10^−6^. Adjustments for multiple testing of all SNVs in a gene with the SMT were done using the BH correction. All rare and (non-causal) common SNVs were incorporated in the analysis.
(PDF)

S11 Table Comparison of power estimates for identifying a causal gene and a causal SNV with a given MAF. Data was generated under the alternative-hypothesis model described in scenarios 13–24 in Table 1 with size *n* = 1,000 for *m* = 10,000 replicates. The nominal *α* was set to 2.5∙10^−6^ for the gene-level evaluation of SMT and SKAT-O (representing the Bonferroni-correction for testing 20,000 genes), cf. Fig 2, and to 10^−7^ for the SNV-level evaluation of SMT (representing the Bonferroni-correction for testing 500,000 SNVs). Adjustments for multiple testing of all SNVs in a gene were done using the BH-correction. The power of the SMT for identifying a causal SNV with a given MAF is based on all causal SNVs in the *m* = 10,000 replicates with the specified MAF. It is estimated by the number of significant causal SNVs (with p-values smaller than 10^−7^ divided by the total number of causal SNVs with the specified MAF. The power for testing singletons and doubletons is even smaller than the power of testing SNVs with MAF = 0.002.
(PDF)

S12 Table Conditional power estimates of the SMT for identifying a causal SNV, given that the gene contains a causal SNV. Data was generated under an alternative-hypothesis model described in scenarios 13–24 in Table 1 with size *n* = 1,000 for *m* = 10,000 replicates. The nominal *α* level was set to 0.002, representing the Bonferroni-correction for testing 25 SNVs in a gene (assuming 500,000 SNVs in total within 20,000 genes). The power of the SMT for identifying a causal SNV with a given MAF is based on all causal SNVs in the *m* = 10,000 replicates with the specified MAF. It is estimated by the number of significant causal SNVs (with p-values smaller than 0.002) divided by the total number of causal SNVs with the specified MAF.
(PDF)

S1 Fig Histogram of the SMT t-test statistic values for singletons, doubletons, and for all SNVs. Datasets were generated from the null model described in scenario 0 in Table 1 with size *n* = 1,000 for *m* = 10,000,000 replicates. The histograms and provided descriptive statistics (mean, standard deviation) are based on the 132,797,000 t-test statistics of all singletons in all replicates (left panel), on the 41,341,000 t-test statistics of all doubletons in all replicates (middle panel), and on the 325,393,000 t-test statistics of all SNVs in all replicates (right panel). Overlaid in a blue line is the empirical estimate of the density function. The theoretical mean and standard deviation (SD) of the t_1000-4_ distribution are 0 and 996/994 = 1.001.
(PDF)

## References

[1] Welter D, MacArthur J, Morales J, Burdett T, Hall P, Junkins H, et al The NHGRI GWAS Catalog, a curated resource of SNP-trait associations. Nucleic Acids Res. 2014; 42:D1001–D1006. doi: 10.1093/nar/gkt1229 24316577 10.1093/nar/gkt1229 PMC3965119

[2] Gorlov IP, Gorlova OY, Frazier ML, Spitz MR, Amos CI. Evolutionary evidence of the effect of rare variants on disease etiology. Clin Genet. 2011; 79:199–206. doi: 10.1111/j.1399-0004.2010.01535.x 20831747 10.1111/j.1399-0004.2010.01535.x PMC3652532

[3] Nelson MR, Wegmann D, Ehm MG, Kessner D, St. Jean P, Verzilli C, et al An abundance of rare functional variants in 202 drug target genes sequenced in 14,002 people. Science. 2012; 337:100–104. doi: 10.1126/science.1217876 22604722 10.1126/science.1217876 PMC4319976

[4] Purcell SM, Moran JL, Fromer M, Ruderfer D, Solovieff N, Roussous P, et al A polygenic burden of rare disruptive mutations in schizophrenia. Nature. 2014; 506:185–190. doi: 10.1038/nature12975 24463508 10.1038/nature12975 PMC4136494

[5] Hunt KA, Mistry V, Bockett NA, Ahmad T, Ban M, Barker JN, et al Negligible impact of rare autoimmune-locus coding-region variants on missing heritability. Nature. 2013; 498:232–235. doi: 10.1038/nature12170 23698362 10.1038/nature12170 PMC3736321

[6] Lohmueller KE, Sparsø T, Li Q, Andersson E, Korneliussen T, Albrechtsen A, et al Whole-exome sequencing of 2,000 Danish individuals and the role of rare coding variants in type 2 diabetes. Am J Hum Genet. 2013; 93:1072–1086. doi: 10.1016/j.ajhg.2013.11.005 24290377 10.1016/j.ajhg.2013.11.005 PMC3852935

[7] UK10K Consortium. The UK10K project identifies rare variants in health and disease. Nature. 2015; 526:82–90. doi: 10.1038/nature14962 26367797 10.1038/nature14962 PMC4773891

[8] Li B, Leal SM. Methods for detecting associations with rare variants for common diseases: application to analysis of sequence data. Am J Hum Genet. 2008; 83:311–321. doi: 10.1016/j.ajhg.2008.06.024 18691683 10.1016/j.ajhg.2008.06.024 PMC2842185

[9] Madsen BE, Browning SR. A groupwise association test for rare mutations using a weighted sum statistic. PLoS Genet. 2009; 5:e1000384 doi: 10.1371/journal.pgen.1000384 19214210 10.1371/journal.pgen.1000384 PMC2633048

[10] Morgenthaler S, Thilly WG. A strategy to discover genes that carry multi-allelic or mono-allelic risk for common diseases: a cohort allelic sums test (CAST). Mutat Res. 2007; 615:28–56. doi: 10.1016/j.mrfmmm.2006.09.003 17101154 10.1016/j.mrfmmm.2006.09.003

[11] Morris AP, Zeggini E. An evaluation of statistical approaches to rare variant analysis in genetic association studies. Genet Epidemiol. 2010; 34:188–193. doi: 10.1002/gepi.20450 19810025 10.1002/gepi.20450 PMC2962811

[12] Chen LS, Hsu L, Gamazon ER, Cox NJ, Nicole DL. An exponential combination procedure for set-based association tests in sequencing studies. Am J Hum Genet. 2012; 91:977–986. doi: 10.1016/j.ajhg.2012.09.017 23159251 10.1016/j.ajhg.2012.09.017 PMC3516612

[13] Lin DY, Tang ZZ. A general framework for detecting disease associations with rare variants in sequencing studies. Am J Hum Genet. 2011; 89:354–367. doi: 10.1016/j.ajhg.2011.07.015 21885029 10.1016/j.ajhg.2011.07.015 PMC3169821

[14] Price AL, Kryukov GV, de Bakker PIW, Purcell SM, Staples J, Wei LJ, et al Pooled association tests for rare variants in exon-resequencing studies. Am J Hum Genet. 2010; 86:832–838. doi: 10.1016/j.ajhg.2010.04.005 20471002 10.1016/j.ajhg.2010.04.005 PMC3032073

[15] Basu S, Pan W. Comparison of statistical tests for disease association with rare variants. Genet Epidemiol. 2011; 35:606–619. doi: 10.1002/gepi.20609 21769936 10.1002/gepi.20609 PMC3197766

[16] Neale BM, Rivas MA, Voight BF, Altshuler D, Devlin B, Orho-Melander M, et al Testing for an unusual distribution of rare variants. PLoS Genet. 2011; 7:e1001322 doi: 10.1371/journal.pgen.1001322 21408211 10.1371/journal.pgen.1001322 PMC3048375

[17] Wu MC, Lee S, Cai T, Li Y, Boehnke M, Lin X. Rare-variant association testing for sequencing data with the sequence kernel association test. Am J Hum Genet. 2011; 89:82–93. doi: 10.1016/j.ajhg.2011.05.029 21737059 10.1016/j.ajhg.2011.05.029 PMC3135811

[18] Derkach A, Lawless JF, Sun L. Robust and powerful tests for rare variants using Fisher’s method to combine evidence of association from two or more complementary tests. Genet Epidemiol. 2013; 37:110–121. doi: 10.1002/gepi.21689 23032573 10.1002/gepi.21689

[19] Lee S, Wu MC, Lin X Optimal tests for rare variant effects in sequencing association studies. Biostatistics. 2012; 13:762–775. doi: 10.1093/biostatistics/kxs014 22699862 10.1093/biostatistics/kxs014 PMC3440237

[20] Pan W, Kim J, Zhang Y, Shen X, Wei P. A powerful and adaptive association test for rare variants. Genetics. 2014; 197:1081–1095. doi: 10.1534/genetics.114.165035 24831820 10.1534/genetics.114.165035 PMC4125385

[21] Sun J, Zheng Y, Hsu L. A unified mixed-effects model for rare-variant association in sequencing studies. Genet Epidemiol. 2013; 37:334–344. doi: 10.1002/gepi.21717 23483651 10.1002/gepi.21717 PMC3740585

[22] Xu C, Ladouceur M, Dastani Z, Richards JB, Ciampi A, Greenwood CMT. Multiple regression methods show great potential for rare variant association tests. PLoS One. 2012; 7:e41694 doi: 10.1371/journal.pone.0041694 22916111 10.1371/journal.pone.0041694 PMC3420665

[23] Kinnamon DD, Hershberger RE, Martin ER. Reconsidering association testing methods using single-variant test statistics as alternatives to pooling tests for sequence data with rare variants. PLoS One. 2012; 7(2):e30238 doi: 10.1371/journal.pone.0030238 22363423 10.1371/journal.pone.0030238 PMC3281828

[24] Xing G, Lin CY, Wooding SP, Xing C. Blindly using Wald’s test can miss rare disease-causal variants in case-control association studies. Ann Hum Genet. 2012; 76:168–177. doi: 10.1111/j.1469-1809.2011.00700.x 22256951 10.1111/j.1469-1809.2011.00700.x

[25] Asimit J, Zeggini E. Rare variant association analysis methods for complex traits. Annu Rev Genet. 2010; 44:293–308. doi: 10.1146/annurev-genet-102209-163421 21047260 10.1146/annurev-genet-102209-163421

[26] Lee S, Abecasis GR, Boehnke M, Lin X. Rare-variant association analysis: study designs and statistical tests. Am J Hum Genet. 2014; 95:5–23. doi: 10.1016/j.ajhg.2014.06.009 24995866 10.1016/j.ajhg.2014.06.009 PMC4085641

[27] Sham PC, Purcell SM. Statistical power and significance testing in large-scale genetic studies. Nat Rev Genet. 2014; 15:335–346. doi: 10.1038/nrg3706 24739678 10.1038/nrg3706

[28] Ladouceur M, Dastani Z, Aulchenko YS, Greenwood CMT, Richards JB. The empirical power of rare variant association methods: results from Sanger sequencing in 1,998 individuals. PLoS Genet. 2012; 8(2):e1002496 doi: 10.1371/journal.pgen.1002496 22319458 10.1371/journal.pgen.1002496 PMC3271058

[29] Saad M, Saint Pierre A, Bohossian N, Macé M, Martinez M. Comparative study of statistical methods for detecting association with rare variants in exome-resequencing data. BMC Proc. 2011; 5 Suppl 9:S33. 10.1186/1753-6561-5-S9-S33 PMC3287869 22373523

[30] Xu C, Ciampi A, Greenwood CMT; The UK10K Consortium. Exploring the potential benefits of stratified false discovery rates for region-based testing of association with rare genetic variation. Front Genet. 2014; 5:1–13. 24523729 10.3389/fgene.2014.00011 PMC3905218

[31] Wang SR, Agarwala V, Flannick J, Chiang CW, Altshuler D; GoT2D Consortium, et al Simulation of Finnish population history, guided by empirical genetic data, to assess power of rare-variant tests in Finland. Am J Hum Genet. 2014; 94:710–720. doi: 10.1016/j.ajhg.2014.03.019 24768551 10.1016/j.ajhg.2014.03.019 PMC4067550

[32] Pan W, Shen X. Adaptive tests for association analysis of rare variants. Genet Epidemiol. 2011; 35:381–388. doi: 10.1002/gepi.20586 21520272 10.1002/gepi.20586 PMC3345534

[33] Chun H, Ballard DH, Cho J, Zhao H. Identification of association between disease and multiple markers via sparse partial least-squares regression. Genet Epidemiol. 2011; 35:479–86. doi: 10.1002/gepi.20596 21678491 10.1002/gepi.20596

[34] Huang J, Wang K, Wei P, Liu X, Liu X, Tan K, et al FLAGS: A flexible and adaptive association test for gene sets using summary statistics. Genetics. 2016; 202:919–929. doi: 10.1534/genetics.115.185009 26773050 10.1534/genetics.115.185009 PMC4788129

[35] Wang X, Morris NJ, Schaid DJ, Elston RC. Power of single- vs. multi-marker tests of association. Genet Epidemiol. 2012; 36:480–487. doi: 10.1002/gepi.21642 22648939 10.1002/gepi.21642 PMC3708310

[36] Benjamini Y, Hochberg Y. Controlling the false discovery rate: a practical and powerful approach to multiple testing. J R Stat Soc B. 1995; 57:289–300.

[37] Lee S, Miropolsky L, Wu MC. SKAT: SNP-Set (Sequence) Kernel Association Test. Version 1.2.1 [R package]. 2016. Available from: https://CRAN.R-project.org/package=SKAT.

[38] Laird NM, Lange C. The fundamentals of modern statistical genetics New York: Springer; 2011.

[39] Knight K. Mathematical statistics Boca Raton: Chapman & Hall/CRC; 1999.

[40] Blangero J, Teslovich TM, Sim X, Almeida MA, Jun G, Dyer TD, et al Omics-squared: human genomic, transcriptomic and phenotypic data for Genetic Analysis Workshop 19. BMC Proc. 2016; 10(Suppl 7): 71–77. doi: 10.1186/s12919-016-0008-y 27980614 10.1186/s12919-016-0008-y PMC5133484

[41] Konigorski S, Yilmaz YE, Pischon T. Genetic association analysis based on a joint model of gene expression and blood pressure. BMC Proc. 2016; 10(Suppl 7): 289–294. doi: 10.1186/s12919-016-0045-6 27980651 10.1186/s12919-016-0045-6 PMC5133480

[42] Ehret GB, Ferreira T, Chasman DI, Jackson AU, Schmidt EM, Johnson T, et al The genetics of blood pressure regulation and its target organs from association studies in 342,415 individuals. Nat Genet. 2016; 48: 1171–1184. doi: 10.1038/ng.3667 27618452 10.1038/ng.3667 PMC5042863

[43] Kato N, Loh M, Takeuchi F, Verweij N, Wang X, Zhang W, et al Trans-ancestry genome-wide association study identifies 12 genetic loci influencing blood pressure and implicates a role for DNA methylation. Nat Genet. 2015; 47: 1282–1293. doi: 10.1038/ng.3405 26390057 10.1038/ng.3405 PMC4719169

[44] Liu C, Kraja AT, Smith JA, Brody JA, Franceschini N, Bis JC, et al Meta-analysis identifies common and rare variants influencing blood pressure and overlapping with metabolic trait loci. Nat Genet. 2016; 48: 1162–1170. doi: 10.1038/ng.3660 27618448 10.1038/ng.3660 PMC5320952

[45] Surendran P, Drenos F, Young R, Warren H, Cook JP, Manning AK, et al Trans-ancestry meta-analyses identify rare and common variants associated with blood pressure and hypertension. Nat Genet. 2016; 48: 1151–1161. doi: 10.1038/ng.3654 27618447 10.1038/ng.3654 PMC5056636

[46] Konigorski S, Yilmaz YE, Bull SB. Bivariate genetic association analysis of systolic and diastolic blood pressure by copula models. BMC Proc. 2014; 8(Suppl 1): S72 doi: 10.1186/1753-6561-8-S1-S72 25519342 10.1186/1753-6561-8-S1-S72 PMC4143670

[47] Sun L, Dimitromanolakis A, Faye LL, Paterson AD, Waggott D; DCCT/EDIC Research Group, et al BR-Squared: a practical solution to the winner’s curse in genome-wide scans. Hum Genet. 2011; 129:545–552. doi: 10.1007/s00439-011-0948-2 21246217 10.1007/s00439-011-0948-2 PMC3074069

