# Supplementary material for: Comparison of single-marker and multi-marker tests in rare variant association studies of quantitative traits
Source: PLoS One. 2017 May 31;12(5):e0178504. doi: 10.1371/journal.pone.0178504 (PMC5451057; doi:10.1371/journal.pone.0178504)
Supplement: S1 Text — (PDF) [file pone.0178504.s001.pdf]

S1 Text. Details of the Analysis of Binary Traits

Simulation Study – Data Generation

For a comparison of the power in studies of quantitative traits and binary traits, case-control phenotypes were generated for the scenarios 1, 4, 7, 8, 12 in Table 1 using a logistic regression model, with the same genetic data (SNVs g_i), the same effect sizes β_i, and the same percentages of causal variants and direction of effects as for quantitative traits. The disease status Y was generated from a logistic regression model with

$$Y|x_1, x_2, g_1, \dots, g_k \sim Bernoulli\big(p(x_1, x_2, g_1, \dots, g_k)\big)$$

where $p(x_1, x_2, g_1, \dots, g_k) = \frac{\exp(0.5x_1+0.5x_2+\sum \beta_i g_i)}{1+\exp(0.5x_1+0.5x_2+\sum \beta_i g_i)}$ for the k SNVs g_i in a gene, $x_1 \sim Bin(0.5)$ which was centered for the data generation, $x_2 \sim N(0,1)$ and $\beta_i = c \cdot |log_{10}(\text{MAF}_i)|$.

Simulation Study – Methods

To evaluate the performance of SMTs, we fitted the logistic regression model of Y,

$$\Pr(Y = 1|x_1, x_2, g_i) = \frac{e^{\gamma_0+\gamma_1 x_1+\gamma_2 x_2+\beta_i g_i}}{1+ e^{\gamma_0+\gamma_1 x_1+\gamma_2 x_2+\beta_i g_i}} \qquad \text{(S1)}$$

separately for each SNV g_i in a gene using the *glm()* function in R (with default settings), obtained the maximum likelihood estimate $\widehat{\beta_i}$ and its standard error estimate $\widehat{SE}\big(\widehat{\beta_i}\big)$, and computed the Wald test for testing $H_{0_i}: \beta_i = 0$. Adjustments for multiple testing of all SNVs in a gene with the SMT were done using the BH correction and the minimum p-value in a gene was extracted for the gene-level evaluation.

For the burden test, we similarly used the *glm()* function in R with default settings to obtain maximum likelihood estimates and to compute Wald tests for testing $H_0: \alpha = 0$, by fitting the logistic regression model

$$\Pr(Y = 1|x_1, x_2, g_1, \dots, g_k) = \frac{e^{\gamma_0+\gamma_1 x_1+\gamma_2 x_2+\alpha \sum g_i}}{1 + e^{\gamma_0+\gamma_1 x_1+\gamma_2 x_2+\alpha \sum g_i}}.$$

For SKAT and SKAT-O, the *SKATBinary* function in the R SKAT package was used with default settings.

Results

Regarding the single-marker approach, while maximum likelihood estimation in the logistic regression model (S1) did not provide valid point estimates and SE estimates for testing singletons and doubletons, the type I errors of the SMT gene-level tests were not inflated and rather very conservative (data not shown). The results of the power comparison are shown in S9 and S10 Tables and indicate that the power of the SMT and MMTs was much lower compared to the analysis of quantitative traits. The decrease was much stronger for the SMT, which had a very low power across all scenarios.
