# Supplementary material for: Comparison of single-marker and multi-marker tests in rare variant association studies of quantitative traits
Source: PLoS One. 2017 May 31;12(5):e0178504. doi: 10.1371/journal.pone.0178504 (PMC5451057; doi:10.1371/journal.pone.0178504)
Supplement: S2 Text — (PDF) [file pone.0178504.s002.pdf]

S2 Text. Details of the Power Comparison for Identifying a Causal Variant and a Causal Gene

S11 Table shows power estimates of SMTs for identifying a causal single variant with a given MAF in comparison to the power for identifying a causal gene. For the interpretation of these results, it should be noted that the power estimates are based on testing different hypotheses and corrected for multiple testing of $20{,}000$ genes in the gene-level analysis and $500{,}000$ SNVs in the variant-level analysis. The power for identifying a single variant is very low for variants with small MAF. When a variant was tested with 10 observed minor alleles (which equals $\mathrm{MAF} = 0.005$ in this study), then the power for identifying the causal SNV increased and was around 20% for larger effect sizes (scenarios 13, 16, 19, 22 in S11 Table) and less than 6% for medium and small effect sizes in the other scenarios.

If SMTs or MMTs are first used to identify genes that are associated with the trait, then SMTs have to be used in a second stage to identify causal SNVs, and the power estimates should be adjusted to account for the follow-up testing. Developing and evaluating the power of such a 2-stage method was not the focus of this study, and as an upper limit to this power, we show conditional power estimates in S12 Table, obtained by applying the SMT in the second stage to test all SNVs in a gene which has been identified as causal in the first stage. The conditional power was on average 13% for singletons and 85% for SNVs with 10 observed minor alleles for larger effect sizes (scenarios 13, 16, 19, 22 in S12 Table), on average 2% and 23% for medium effect sizes (scenarios 14, 17, 20, 23 in S12 Table) and on average 1% and 8% (scenarios 15, 18, 21, 24 in S12 Table) for smaller effect sizes.
