# Supplementary material for: Comparison of single-marker and multi-marker tests in rare variant association studies of quantitative traits
Source: PLoS One. 2017 May 31;12(5):e0178504. doi: 10.1371/journal.pone.0178504 (PMC5451057; doi:10.1371/journal.pone.0178504)
Supplement: S1 Table — Datasets were generated from the null model described in scenario 0 in Table 1 with size n = 1,000 for m = 10,000,000 replicates. The table shows the mean and standard deviation of the SNV effect estimates and the mean of the corresponding standard error estimates, for the 132,797,000 singletons in all replicates, the 41,341,000 doubletons in all replicates, and the 2,985,000 SNVs with 10 observed minor alleles (minor allele count MAC = 10). (PDF) [file pone.0178504.s003.pdf]

Variant	$mean(\widehat{\beta_\iota})$	$SD(\widehat{\beta_\iota})$	$mean(\widehat{SE}_{\widehat{\beta_\iota}})$
Singleton	$1.4 \cdot 10^{-4}$	1.00	1.00
Doubleton	$1.0 \cdot 10^{-4}$	0.71	0.71
SNV with $MAC = 10$	$1.0 \cdot 10^{-4}$	0.32	0.32

S1 Table. Descriptive statistics of (restricted) maximum likelihood estimates from linear regression model under the null hypothesis.

Datasets were generated from the null model described in scenario 0 in Table 1 with size $\mathrm{n} = 1{,}000$ for $\mathrm{m} = 10{,}000{,}000$ replicates. The table shows the mean and standard deviation of the SNV effect estimates and the mean of the corresponding standard error estimates, for the $132{,}797{,}000$ singletons in all replicates, the $41{,}341{,}000$ doubletons in all replicates, and the 2,985,000 SNVs with 10 observed minor alleles (minor allele count $MAC = 10$).
