# Supplementary material for: Comparison of single-marker and multi-marker tests in rare variant association studies of quantitative traits
Source: PLoS One. 2017 May 31;12(5):e0178504. doi: 10.1371/journal.pone.0178504 (PMC5451057; doi:10.1371/journal.pone.0178504)
Supplement: S2 Table — Data was generated from the null model with size n = 1,000 for m = 10,000,000 replicates, with the error distribution of the phenotype given covariates chosen to be from a standard normal (cf. Table 1), t4, t8, or log standard normal distribution. All approaches were computed as described in the main manuscript assuming normally distributed error terms. Adjustments for multiple testing of all SNVs in a gene with the SMT were done using the BH and Bonferroni corrections. The type I error estimates are based on analyses using all rare and (non-causal) common SNVs in a gene, as well as restricting the analysis to SNVs with at least 2 (MAC>1) or 3 (MAC>2) observed minor alleles for the SMT. For other nominal levels (e.g. 10−2, 10−3, 10−4, 10−5, 2.5∙10−6, SKAT, SKAT-O and the SMT showed similar results and inflated type I errors, with a much higher inflation for the SMT. (PDF) [file pone.0178504.s004.pdf]

Error Distribution	SKAT	SKAT-O	Burden	SMT – BH	SMT – Bonferroni	SMT – Bonferroni MAC>1	SMT – Bonferroni MAC>2
$N(0,1)$	$4.94 \cdot 10^{-2}$	$5.22 \cdot 10^{-2}$	$5.01 \cdot 10^{-2}$	$4.39 \cdot 10^{-2}$	$4.93 \cdot 10^{-2}$	--	--
$t_{(8)}$	$5.17 \cdot 10^{-2}$	$5.39 \cdot 10^{-2}$	$5.00 \cdot 10^{-2}$	$12.68 \cdot 10^{-2}$	$12.01 \cdot 10^{-2}$	$6.53 \cdot 10^{-2}$	$5.09 \cdot 10^{-2}$
$t_{(4)}$	$6.13 \cdot 10^{-2}$	$6.09 \cdot 10^{-2}$	$5.00 \cdot 10^{-2}$	$21.30 \cdot 10^{-2}$	$20.61 \cdot 10^{-2}$	$10.64 \cdot 10^{-2}$	$7.57 \cdot 10^{-2}$
$Log\ N(0,1)$	$8.84 \cdot 10^{-2}$	$7.95 \cdot 10^{-2}$	$4.96 \cdot 10^{-2}$	$33.63 \cdot 10^{-2}$	$33.05 \cdot 10^{-2}$	$18.88 \cdot 10^{-2}$	$13.47 \cdot 10^{-2}$

S2 Table. Empirical type I error estimates of the SMT and MMTs for the nominal level $\alpha = 0.05$ when the distribution of the phenotype is misspecified.

Data was generated from the null model with size $n = 1{,}000$ for $m = 10{,}000{,}000$ replicates, with the error distribution of the phenotype given covariates chosen to be from a standard normal (cf. Table 1), $t_{(4)}, t_{(8)}$, or log standard normal distribution. All approaches were computed as described in the main manuscript assuming normally distributed error terms. Adjustments for multiple testing of all SNVs in a gene with the SMT were done using the BH and Bonferroni corrections. The type I error estimates are based on analyses using all rare and (non-causal) common SNVs in a gene, as well as restricting the analysis to SNVs with at least 2 (“MAC>1”) or 3 (“MAC>2”) observed minor alleles for the SMT. For other nominal levels (e.g. $10^{-2}, 10^{-3}, 10^{-4}, 10^{-5}, 2.5 \cdot 10^{-6}$), SKAT, SKAT-O and the SMT showed similar results and inflated type I errors, with a much higher inflation for the SMT.
