# Supplementary material for: Comparison of single-marker and multi-marker tests in rare variant association studies of quantitative traits
Source: PLoS One. 2017 May 31;12(5):e0178504. doi: 10.1371/journal.pone.0178504 (PMC5451057; doi:10.1371/journal.pone.0178504)
Supplement: S3 Table — Data was generated under the alternative-hypothesis model described in scenarios 1–36 in Table 1 with size n = 1,000 for m = 10,000 replicates. The nominal α level was set to 0.05. Adjustments for multiple testing of all SNVs in a gene with the SMT were done using the BH correction. The power results are provided for analyses using all rare and (non-causal) common SNVs in a gene, and for using all rare SNVs in a gene by excluding the common SNVs from the analysis. (PDF) [file pone.0178504.s005.pdf]

Scenario	Using all rare and common SNVs				Using all rare SNVs			
	**SKAT**	**SKAT-O**	**Burden**	**SMT**	**SKAT**	**SKAT-O**	**Burden**	**SMT**
1	0.341	0.337	0.107	0.546	0.358	0.357	0.242	0.563
2	0.199	0.187	0.080	0.192	0.209	0.204	0.140	0.200
3	0.127	0.124	0.062	0.100	0.130	0.130	0.099	0.106
4	0.532	0.534	0.170	0.736	0.553	0.559	0.414	0.752
5	0.299	0.293	0.102	0.282	0.316	0.317	0.230	0.297
6	0.168	0.170	0.076	0.130	0.182	0.185	0.148	0.137
7	0.764	0.783	0.290	0.907	0.792	0.815	0.698	0.917
8	0.477	0.492	0.159	0.440	0.507	0.532	0.440	0.458
9	0.289	0.311	0.115	0.209	0.308	0.337	0.284	0.220
10	0.986	0.996	0.583	0.997	0.993	0.999	0.992	0.998
11	0.825	0.895	0.352	0.730	0.861	0.928	0.896	0.755
12	0.580	0.695	0.242	0.407	0.619	0.752	0.721	0.433
13	0.352	0.346	0.113	0.554	0.368	0.361	0.245	0.570
14	0.188	0.180	0.075	0.187	0.197	0.193	0.133	0.196
15	0.113	0.108	0.060	0.094	0.119	0.113	0.089	0.099
16	0.516	0.497	0.136	0.727	0.540	0.522	0.310	0.744
17	0.300	0.277	0.086	0.280	0.318	0.296	0.170	0.293
18	0.165	0.151	0.070	0.132	0.177	0.163	0.107	0.139
19	0.761	0.747	0.227	0.903	0.785	0.777	0.523	0.913
20	0.473	0.454	0.131	0.432	0.503	0.489	0.310	0.456
21	0.282	0.269	0.093	0.198	0.297	0.290	0.196	0.211
22	0.981	0.981	0.410	0.996	0.988	0.989	0.837	0.997
23	0.806	0.811	0.229	0.713	0.842	0.849	0.625	0.737
24	0.541	0.549	0.163	0.374	0.583	0.602	0.445	0.399
25	0.352	0.335	0.098	0.543	0.367	0.351	0.200	0.561
26	0.195	0.179	0.075	0.188	0.206	0.188	0.117	0.199
27	0.125	0.114	0.058	0.093	0.128	0.117	0.078	0.098
28	0.528	0.499	0.132	0.732	0.548	0.519	0.274	0.749
29	0.295	0.270	0.084	0.286	0.314	0.292	0.159	0.303
30	0.163	0.148	0.063	0.125	0.172	0.158	0.105	0.134
31	0.755	0.727	0.187	0.903	0.782	0.752	0.390	0.914
32	0.460	0.422	0.103	0.416	0.488	0.452	0.224	0.438
33	0.270	0.235	0.076	0.184	0.289	0.255	0.136	0.200
34	0.981	0.973	0.280	0.995	0.986	0.981	0.577	0.997
35	0.801	0.763	0.156	0.692	0.838	0.802	0.346	0.718
36	0.526	0.472	0.111	0.352	0.566	0.514	0.233	0.376

S3 Table. Power estimates of the SMT and MMTs under the nominal α level of 0.05.

Data was generated under the alternative-hypothesis model described in scenarios 1-36 in Table 1 with size $n = 1{,}000$ for $m = 10{,}000$ replicates. The nominal α level was set to 0.05. Adjustments for multiple testing of all SNVs in a gene with the SMT were done using the BH correction. The power results are provided for analyses using all rare and (non-causal) common SNVs in a gene, and for using all rare SNVs in a gene by excluding the common SNVs from the analysis.
