# Supplementary material for: Comparison of single-marker and multi-marker tests in rare variant association studies of quantitative traits
Source: PLoS One. 2017 May 31;12(5):e0178504. doi: 10.1371/journal.pone.0178504 (PMC5451057; doi:10.1371/journal.pone.0178504)
Supplement: S4 Table — Data was generated under the alternative-hypothesis model described in scenarios 1–36 in Table 1 with size n = 1,000 for m = 10,000 replicates. The nominal α level was set to 2.5∙10−6. Adjustments for multiple testing of all SNVs in a gene with the SMT were done using the BH correction. Power results are provided for analyses using all rare and (non-causal) common SNVs in a gene, and for using all rare SNVs in a gene by excluding the common SNVs from the analysis. (PDF) [file pone.0178504.s006.pdf]

Scenario	Using all rare and common SNVs				Using all rare SNVs			
	SKAT	SKAT-O	Burden	SMT	SKAT	SKAT-O	Burden	SMT
1	0.118	0.111	0.012	0.156	0.129	0.122	0.050	0.159
2	0.007	0.008	0.002	0.005	0.008	0.009	0.004	0.005
3	0.001	0.001	<0.001	0.001	0.001	0.001	0.001	0.001
4	0.223	0.215	0.024	0.263	0.243	0.235	0.121	0.268
5	0.021	0.021	0.004	0.014	0.022	0.022	0.015	0.014
6	0.004	0.004	<0.001	0.002	0.003	0.004	0.002	0.002
7	0.410	0.414	0.065	0.422	0.443	0.451	0.306	0.428
8	0.063	0.071	0.012	0.043	0.067	0.077	0.056	0.045
9	0.014	0.016	0.003	0.009	0.015	0.018	0.014	0.009
10	0.797	0.867	0.207	0.698	0.849	0.927	0.874	0.707
11	0.248	0.350	0.070	0.155	0.273	0.421	0.388	0.156
12	0.093	0.128	0.036	0.059	0.095	0.147	0.139	0.060
13	0.124	0.117	0.013	0.158	0.134	0.128	0.053	0.162
14	0.007	0.006	0.001	0.005	0.007	0.007	0.003	0.005
15	0.001	0.001	0	0.001	0.001	0.001	<0.001	0.001
16	0.210	0.200	0.016	0.250	0.227	0.216	0.068	0.256
17	0.015	0.014	0.001	0.008	0.016	0.015	0.006	0.008
18	0.002	0.002	<0.001	0.001	0.002	0.002	0.001	0.001
19	0.393	0.380	0.038	0.406	0.425	0.415	0.178	0.414
20	0.050	0.048	0.007	0.029	0.055	0.054	0.028	0.031
21	0.011	0.015	0.001	0.006	0.011	0.012	0.007	0.006
22	0.764	0.771	0.108	0.667	0.815	0.828	0.522	0.678
23	0.203	0.212	0.038	0.110	0.228	0.246	0.159	0.111
24	0.057	0.063	0.015	0.035	0.060	0.067	0.053	0.035
25	0.123	0.114	0.009	0.161	0.137	0.128	0.035	0.164
26	0.007	0.006	<0.001	0.004	0.008	0.007	0.002	0.005
27	<0.001	<0.001	0	<0.001	0.001	<0.001	<0.001	<0.001
28	0.212	0.199	0.015	0.252	0.231	0.217	0.060	0.257
29	0.016	0.014	0.001	0.009	0.018	0.015	0.005	0.009
30	0.002	0.001	<0.001	0.001	0.001	0.001	0.001	0.001
31	0.393	0.375	0.025	0.403	0.424	0.405	0.105	0.411
32	0.039	0.034	0.004	0.020	0.045	0.038	0.013	0.021
33	0.006	0.006	0	0.003	0.006	0.006	0.003	0.003
34	0.746	0.732	0.047	0.661	0.792	0.782	0.207	0.671
35	0.166	0.147	0.009	0.068	0.187	0.165	0.042	0.070
36	0.035	0.031	0.004	0.016	0.038	0.033	0.012	0.017

S4 Table. Power estimates of the SMT and MMTs under the nominal α level of $2.5 \cdot 10^{-6}$.

Data was generated under the alternative-hypothesis model described in scenarios 1-36 in Table 1 with size $n = 1{,}000$ for $m = 10{,}000$ replicates. The nominal α level was set to $2.5 \cdot 10^{-6}$. Adjustments for multiple testing of all SNVs in a gene with the SMT were done using the BH correction. Power results are provided for analyses using all rare and (non-causal) common SNVs in a gene, and for using all rare SNVs in a gene by excluding the common SNVs from the analysis.
