# Supplementary material for: Comparison of single-marker and multi-marker tests in rare variant association studies of quantitative traits
Source: PLoS One. 2017 May 31;12(5):e0178504. doi: 10.1371/journal.pone.0178504 (PMC5451057; doi:10.1371/journal.pone.0178504)
Supplement: S5 Table — Data was generated under the alternative-hypothesis model described in scenarios 1–12 in Table 1 with size n = 5,000 for m = 10,000 replicates. The genes analyzed in the replicates included on average 58 SNVs. The nominal α level was set to 2.5∙10−6. Adjustments for multiple testing of all SNVs in a gene with the SMT were done using the BH correction. Power results are provided for analyses using all rare and (non-causal) common SNVs in a gene, and for using all rare SNVs in a gene by excluding the common SNVs from the analysis. (PDF) [file pone.0178504.s007.pdf]

Scenario	Using all rare and common SNVs				Using all rare SNVs			
	SKAT	SKAT-O	Burden	SMT	SKAT	SKAT-O	Burden	SMT
1	0.276	0.271	0.063	0.558	0.282	0.280	0.173	0.561
2	0.153	0.148	0.027	0.195	0.160	0.155	0.077	0.196
3	0.060	0.058	0.011	0.047	0.062	0.061	0.029	0.048
4	0.462	0.463	0.117	0.774	0.473	0.480	0.341	0.777
5	0.269	0.265	0.053	0.315	0.279	0.276	0.162	0.318
6	0.117	0.116	0.024	0.088	0.124	0.123	0.075	0.090
7	0.722	0.764	0.243	0.934	0.737	0.792	0.677	0.936
8	0.484	0.497	0.109	0.517	0.505	0.524	0.387	0.521
9	0.252	0.266	0.063	0.167	0.269	0.290	0.208	0.170
10	0.984	0.999	0.583	0.999	0.990	1	0.997	1
11	0.862	0.938	0.309	0.813	0.883	0.965	0.931	0.816
12	0.632	0.765	0.178	0.374	0.664	0.827	0.771	0.379

S5 Table. Power estimates of the SMT and MMTs under the nominal α level of $2.5 \cdot 10^{-6}$, for a sample size of $5{,}000$ and genes including 58 SNVs on average.

Data was generated under the alternative-hypothesis model described in scenarios 1-12 in Table 1 with size $n = 5{,}000$ for $m = 10{,}000$ replicates. The analyzed genes in the replicates included on average 58 SNVs. The nominal α level was set to $2.5 \cdot 10^{-6}$. Adjustments for multiple testing of all SNVs in a gene with the SMT were done using the BH correction. Power results are provided for analyses using all rare and (non-causal) common SNVs in a gene, and for using all rare SNVs in a gene by excluding the common SNVs from the analysis.
