# Supplementary material for: Comparison of single-marker and multi-marker tests in rare variant association studies of quantitative traits
Source: PLoS One. 2017 May 31;12(5):e0178504. doi: 10.1371/journal.pone.0178504 (PMC5451057; doi:10.1371/journal.pone.0178504)
Supplement: S6 Table — Data was generated under the alternative-hypothesis model described in scenarios 1–12 in Table 1 with size n = 5,000 for m = 10,000 replicates. The genes analyzed in the replicates included on average 572 SNVs. The nominal α level was set to 2.5∙10−6. Adjustments for multiple testing of all SNVs in a gene with the SMT were done using the BH correction. Power results are provided for analyses using all rare and (non-causal) common SNVs in a gene, and for using all rare SNVs in a gene by excluding the common SNVs from the analysis. (PDF) [file pone.0178504.s008.pdf]

Scenario	Using all rare and common SNVs				Using all rare SNVs			
	SKAT	SKAT-O	Burden	SMT	SKAT	SKAT-O	Burden	SMT
1	0.861	0.865	0.304	0.998	0.872	0.876	0.724	0.998
2	0.622	0.615	0.191	0.782	0.664	0.660	0.500	0.784
3	0.387	0.391	0.133	0.335	0.414	0.425	0.329	0.339
4	0.989	0.992	0.468	1	0.992	0.995	0.9630	1
5	0.887	0.895	0.304	0.936	0.915	0.930	0.854	0.937
6	0.694	0.717	0.241	0.605	0.737	0.775	0.692	0.608
7	1	1	0.681	1	1	1	1	1
8	0.994	0.998	0.480	0.994	0.997	1	0.997	0.994
9	0.944	0.971	0.364	0.874	0.971	0.991	0.982	0.874
10	1	1	0.877	1	1	1	1	1
11	1	1	0.788	1	1	1	1	1
12	1	1	0.679	0.998	1	1	1	0.998

S6 Table. Power estimates of the SMT and MMTs under the nominal α level of $2.5 \cdot 10^{-6}$, for a sample size of $5{,}000$ and genes including 572 SNVs on average.

Data was generated under the alternative-hypothesis model described in scenarios 1-12 in Table 1 with size $n = 5{,}000$ for $m = 10{,}000$ replicates. The analyzed genes in the replicates included on average 572 SNVs. The nominal α level was set to $2.5 \cdot 10^{-6}$. Adjustments for multiple testing of all SNVs in a gene with the SMT were done using the BH correction. Power results are provided for analyses using all rare and (non-causal) common SNVs in a gene, and for using all rare SNVs in a gene by excluding the common SNVs from the analysis.
