# Supplementary material for: Comparison of single-marker and multi-marker tests in rare variant association studies of quantitative traits
Source: PLoS One. 2017 May 31;12(5):e0178504. doi: 10.1371/journal.pone.0178504 (PMC5451057; doi:10.1371/journal.pone.0178504)
Supplement: S7 Table — As genetic data, the available genotypes of the 67 SNVs of n = 2,000 individuals in the ‘SKAT.example’ data in the SKAT R package were used in each replicate. Data was generated under the alternative-hypothesis model described in scenarios 1, 4, 7, 8, 12 in Table 1 with size n = 2,000 for m = 10,000 replicates. The nominal α level was set to 2.5∙10−6. Adjustments for multiple testing of all SNVs in a gene with the SMT were done using the BH correction. Power results are provided for analyses using all rare and (non-causal) common SNVs in a gene. (PDF) [file pone.0178504.s009.pdf]

Scenario	SKAT	SKAT-O	Burden	SMT
1	0.363	0.350	0.002	0.515
4	0.565	0.554	0.006	0.715
7	0.860	0.864	0.041	0.928
8	0.388	0.406	0.002	0.190
12	0.531	0.733	0.002	0.231

S7 Table. Power estimates of the SMT and MMTs under the nominal α level of $2.5 \cdot 10^{-6}$, for a sample size of $2{,}000$ and genes including 67 SNVs.

As genetic data, the available genotypes of the 67 SNVs of $\mathrm{n} = 2{,}000$ individuals in the 'SKAT.example' data in the SKAT R package were used in each replicate. Data was generated under the alternative-hypothesis model described in scenarios 1, 4, 7, 8, 12 in Table 1 with size $\mathrm{n} = 2{,}000$ for $\mathrm{m} = 10{,}000$ replicates. The nominal α level was set to $2.5 \cdot 10^{-6}$. Adjustments for multiple testing of all SNVs in a gene with the SMT were done using the BH correction. Power results are provided for analyses using all rare and (non-causal) common SNVs in a gene.
