# Supplementary material for: Comparison of single-marker and multi-marker tests in rare variant association studies of quantitative traits
Source: PLoS One. 2017 May 31;12(5):e0178504. doi: 10.1371/journal.pone.0178504 (PMC5451057; doi:10.1371/journal.pone.0178504)
Supplement: S8 Table — Data was generated under the alternative-hypothesis model described in scenarios 1–12 in Table 1 with size n = 1,000 for m = 10,000 replicates. The nominal α level was set to 2.5∙10−6. Adjustments for multiple testing of all SNVs in a gene with the SMT were done using the BH or the Bonferroni correction. Power results are provided for analyses using all rare and (non-causal) common SNVs in a gene. (PDF) [file pone.0178504.s010.pdf]

| Scenario | SKAT | SKAT-O | Burden | SMT – BH | SMT – Bonferroni |
|---|---|---|---|---|---|
| 1 | 0.118 | 0.111 | 0.012 | 0.156 | 0.153 |
| 2 | 0.007 | 0.008 | 0.002 | 0.005 | 0.004 |
| 3 | 0.001 | 0.001 | <0.001 | 0.001 | <0.001 |
| 4 | 0.223 | 0.215 | 0.024 | 0.263 | 0.259 |
| 5 | 0.021 | 0.021 | 0.004 | 0.014 | 0.011 |
| 6 | 0.004 | 0.004 | <0.001 | 0.002 | 0.002 |
| 7 | 0.410 | 0.414 | 0.065 | 0.422 | 0.415 |
| 8 | 0.063 | 0.071 | 0.012 | 0.043 | 0.040 |
| 9 | 0.014 | 0.016 | 0.003 | 0.009 | 0.007 |
| 10 | 0.797 | 0.867 | 0.207 | 0.698 | 0.692 |
| 11 | 0.248 | 0.350 | 0.070 | 0.155 | 0.147 |
| 12 | 0.093 | 0.128 | 0.036 | 0.059 | 0.053 |

S8 Table. Power estimates of the SMT and MMTs under the nominal $\alpha$ level of $2.5 \cdot 10^{-6}$ to compare the BH and Bonferroni correction for SMTs.

Data was generated under the alternative-hypothesis model described in scenarios 1-12 in Table 1 with size $n = 1{,}000$ for $m = 10{,}000$ replicates. The nominal $\alpha$ level was set to $2.5 \cdot 10^{-6}$. Adjustments for multiple testing of all SNVs in a gene with the SMT were done using the BH or the Bonferroni correction. Power results are provided for analyses using all rare and (non-causal) common SNVs in a gene.
