# Supplementary material for: Comparison of single-marker and multi-marker tests in rare variant association studies of quantitative traits
Source: PLoS One. 2017 May 31;12(5):e0178504. doi: 10.1371/journal.pone.0178504 (PMC5451057; doi:10.1371/journal.pone.0178504)
Supplement: S9 Table — Case-control data was generated under the alternative-hypothesis model described in scenarios 1, 4, 7, 8, 12 in Table 1 with size n = 1,000 for m = 10,000 replicates. The nominal α level was set to 0.05. Adjustments for multiple testing of all SNVs in a gene with the SMT were done using the BH correction. All rare and (non-causal) common SNVs were incorporated in the analysis. (PDF) [file pone.0178504.s011.pdf]

Scenario	SKAT	SKAT-O	Burden	SMT
1	0.166	0.157	0.070	0.062
4	0.247	0.238	0.086	0.097
7	0.397	0.408	0.139	0.162
8	0.164	0.178	0.083	0.043
12	0.200	0.270	0.112	0.051

S9 Table. Power estimates of the SMT and MMTs for analyzing a binary trait under the nominal α level of 0.05.

Case-control data was generated under the alternative-hypothesis model described in scenarios 1, 4, 7, 8, 12 in Table 1 with size $n = 1{,}000$ for $m = 10{,}000$ replicates. The nominal α level was set to 0.05. Adjustments for multiple testing of all SNVs in a gene with the SMT were done using the BH correction. All rare and (non-causal) common SNVs were incorporated in the analysis.
