# Supplementary material for: Comparison of single-marker and multi-marker tests in rare variant association studies of quantitative traits
Source: PLoS One. 2017 May 31;12(5):e0178504. doi: 10.1371/journal.pone.0178504 (PMC5451057; doi:10.1371/journal.pone.0178504)
Supplement: S10 Table — Case-control data was generated under the alternative-hypothesis model described in scenarios 1, 4, 7, 8, 12 in Table 1 with size n = 1,000 for m = 10,000 replicates for a binary trait. The nominal α level was set to 2.5∙10−6. Adjustments for multiple testing of all SNVs in a gene with the SMT were done using the BH correction. All rare and (non-causal) common SNVs were incorporated in the analysis. (PDF) [file pone.0178504.s012.pdf]

Scenario	SKAT	SKAT-O	Burden	SMT
1	0.003	0.003	<0.001	0
4	0.009	0.009	0.001	0
7	0.029	0.032	0.005	<0.001
8	0.002	0.002	<0.001	0
12	0.003	0.005	<0.001	0

S10 Table. Power estimates of the SMT and MMTs for analyzing a binary trait under the nominal α level of $2.5 \cdot 10^{-6}$.

Case-control data was generated under the alternative-hypothesis model described in scenarios 1, 4, 7, 8, 12 in Table 1 with size $n = 1{,}000$ for $m = 10{,}000$ replicates for a binary trait. The nominal α level was set to $2.5 \cdot 10^{-6}$. Adjustments for multiple testing of all SNVs in a gene with the SMT were done using the BH correction. All rare and (non-causal) common SNVs were incorporated in the analysis.
