# Supplementary material for: Comparison of single-marker and multi-marker tests in rare variant association studies of quantitative traits
Source: PLoS One. 2017 May 31;12(5):e0178504. doi: 10.1371/journal.pone.0178504 (PMC5451057; doi:10.1371/journal.pone.0178504)
Supplement: S11 Table — Data was generated under the alternative-hypothesis model described in scenarios 13–24 in Table 1 with size n = 1,000 for m = 10,000 replicates. The nominal α was set to 2.5∙10−6 for the gene-level evaluation of SMT and SKAT-O (representing the Bonferroni-correction for testing 20,000 genes), cf. Fig 2, and to 10−7 for the SNV-level evaluation of SMT (representing the Bonferroni-correction for testing 500,000 SNVs). Adjustments for multiple testing of all SNVs in a gene were done using the BH-correction. The power of the SMT for identifying a causal SNV with a given MAF is based on all causal SNVs in the m = 10,000 replicates with the specified MAF. It is estimated by the number of significant causal SNVs (with p-values smaller than 10−7 divided by the total number of causal SNVs with the specified MAF. The power for testing singletons and doubletons is even smaller than the power of testing SNVs with MAF = 0.002. (PDF) [file pone.0178504.s013.pdf]

Scenario	Gene-level		SNV-level	
	SKAT-O	SMT	SMT for SNVs with $\mathbf{MAF = 0.002}$	SMT for SNVs with $\mathbf{MAF = 0.005}$
13	0.117	0.158	0.022	0.188
14	0.006	0.005	0	0
15	0.001	0.001	0	0.010
16	0.200	0.250	0.016	0.220
17	0.014	0.008	0	0.006
18	0.002	0.001	0	0
19	0.380	0.406	0.022	0.203
20	0.048	0.029	0	0.034
21	0.015	0.006	0	0.005
22	0.771	0.667	0.023	0.225
23	0.212	0.110	<0.001	0.055
24	0.063	0.035	0	0.010

S11 Table. Comparison of power estimates for identifying a causal gene and a causal SNV with a given MAF.

Data was generated under the alternative-hypothesis model described in scenarios 13-24 in Table 1 with size $\mathrm{n} = 1{,}000$ for $\mathrm{m} = 10{,}000$ replicates. The nominal α was set to $2.5 \cdot 10^{-6}$ for the gene-level evaluation of SMT and SKAT-O (representing the Bonferroni-correction for testing $20{,}000$ genes), cf. Fig 2, and to 10^{-7} for the SNV-level evaluation of SMT (representing the Bonferroni-correction for testing $500{,}000$ SNVs). Adjustments for multiple testing of all SNVs in a gene were done using the BH-correction. The power of the SMT for identifying a causal SNV with a given MAF is based on all causal SNVs in the $\mathrm{m} = 10{,}000$ replicates with the specified MAF. It is estimated by the number of significant causal SNVs (with p-values smaller than 10^{-7}) divided by the total number of causal SNVs with the specified MAF. The power for testing singletons and doubletons is even smaller than the power of testing SNVs with $MAF = 0.002$.
