# Supplementary material for: Comparison of single-marker and multi-marker tests in rare variant association studies of quantitative traits
Source: PLoS One. 2017 May 31;12(5):e0178504. doi: 10.1371/journal.pone.0178504 (PMC5451057; doi:10.1371/journal.pone.0178504)
Supplement: S12 Table — Data was generated under an alternative-hypothesis model described in scenarios 13–24 in Table 1 with size n = 1,000 for m = 10,000 replicates. The nominal α level was set to 0.002, representing the Bonferroni-correction for testing 25 SNVs in a gene (assuming 500,000 SNVs in total within 20,000 genes). The power of the SMT for identifying a causal SNV with a given MAF is based on all causal SNVs in the m = 10,000 replicates with the specified MAF. It is estimated by the number of significant causal SNVs (with p-values smaller than 0.002) divided by the total number of causal SNVs with the specified MAF. (PDF) [file pone.0178504.s014.pdf]

Scenario	SNV with MAF $= 0.0005$	SNV with MAF $= 0.001$	SNV with MAF $= 0.002$	SNV with MAF $= 0.005$
13	0.133	0.280	0.555	0.875
14	0.020	0.033	0.055	0.200
15	0.008	0.011	0.022	0.039
16	0.131	0.283	0.542	0.873
17	0.015	0.032	0.072	0.179
18	0.009	0.008	0.022	0.052
19	0.127	0.268	0.506	0.859
20	0.018	0.033	0.069	0.290
21	0.008	0.010	0.021	0.082
22	0.114	0.237	0.461	0.801
23	0.021	0.037	0.072	0.267
24	0.009	0.014	0.025	0.158

S12 Table. Conditional power estimates of the SMT for identifying a causal SNV, given that the gene contains a causal SNV.

Data was generated under an alternative-hypothesis model described in scenarios 13-24 in Table 1 with size $n = 1{,}000$ for $m = 10{,}000$ replicates. The nominal α level was set to 0.002, representing the Bonferroni-correction for testing 25 SNVs in a gene (assuming $500{,}000$ SNVs in total within $20{,}000$ genes). The power of the SMT for identifying a causal SNV with a given MAF is based on all causal SNVs in the $m = 10{,}000$ replicates with the specified MAF. It is estimated by the number of significant causal SNVs (with p-values smaller than 0.002) divided by the total number of causal SNVs with the specified MAF.
