# Supplementary material for: Comparison of single-marker and multi-marker tests in rare variant association studies of quantitative traits
Source: PLoS One. 2017 May 31;12(5):e0178504. doi: 10.1371/journal.pone.0178504 (PMC5451057; doi:10.1371/journal.pone.0178504)
Supplement: S1 Fig — Datasets were generated from the null model described in scenario 0 in Table 1 with size n = 1,000 for m = 10,000,000 replicates. The histograms and provided descriptive statistics (mean, standard deviation) are based on the 132,797,000 t-test statistics of all singletons in all replicates (left panel), on the 41,341,000 t-test statistics of all doubletons in all replicates (middle panel), and on the 325,393,000 t-test statistics of all SNVs in all replicates (right panel). Overlaid in a blue line is the empirical estimate of the density function. The theoretical mean and standard deviation (SD) of the t1000-4 distribution are 0 and 996/994 = 1.001. (PDF) [file pone.0178504.s015.pdf]

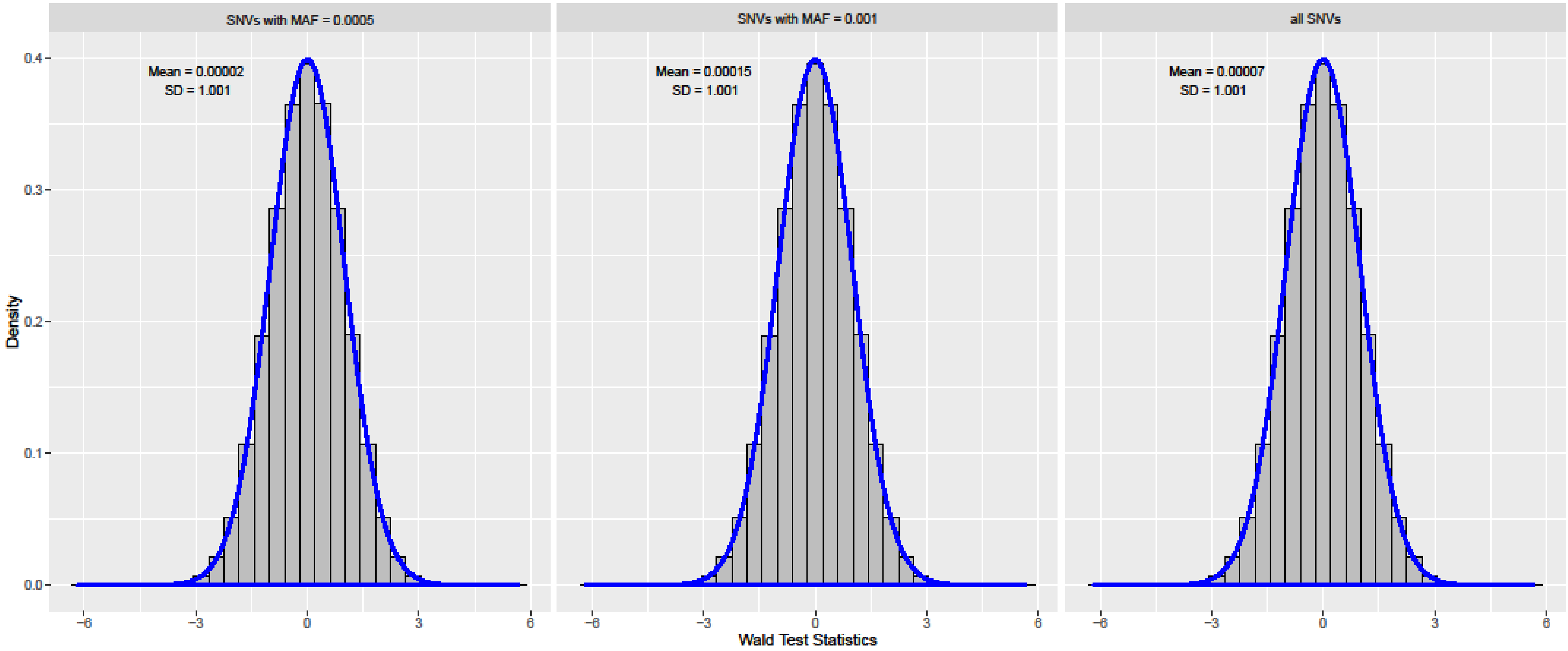

S1 Fig. Histogram of the SMT t-test statistic values for singletons, doubletons, and for all SNVs.

Datasets were generated from the null model described in scenario 0 in Table 1 with size $\mathrm{n} = 1{,}000$ for $\mathrm{m} = 10{,}000{,}000$ replicates. The histograms and provided descriptive statistics (mean, standard deviation) are based on the $132{,}797{,}000$ t-test statistics of all singletons in all replicates (left panel), on the $41{,}341{,}000$ t-test statistics of all doubletons in all replicates (middle panel), and on the $325{,}393{,}000$ t-test statistics of all SNVs in all replicates (right panel). Overlaid in a blue line is the empirical estimate of the density function. The theoretical mean and standard deviation (SD) of the t_{1000-4} distribution are 0 and $996/994 = 1.001$.
